# Nanomaterials Trigger Functional Anti‐Tumoral Responses in Primary Human Immune Cells

**DOI:** 10.1002/advs.202505729

**Published:** 2025-07-12

**Authors:** Vincent Mittelheisser, Olivier Lefebvre, Mainak Banerjee, Shayamita Ghosh, Amandine Dupas, Marie‐Charlotte Diringer, Juliette Blumberger, Louis Bochler, Sébastien Harlepp, Annabel Larnicol, Angélique Pichot, Tristan Stemmelen, Anne Molitor, Chloé Moritz, Christine Carapito, Raphaël Carapito, Loïc J. Charbonnière, François Lux, Olivier Tillement, Jacky G. Goetz, Alexandre Detappe

**Affiliations:** ^1^ Tumor Biomechanics lab Strasbourg 67000 France; ^2^ INSERM UMR_S1109 Strasbourg 67000 France; ^3^ Université de Strasbourg Strasbourg 67000 France; ^4^ Fédération de Médecine Translationnelle de Strasbourg (FMTS) Strasbourg 67000 France; ^5^ Équipe Labellisée Ligue Contre le Cancer Strasbourg 67000 France; ^6^ Nanotranslational lab Institut de cancérologie Strasbourg Europe Strasbourg 67033 France; ^7^ Strasbourg Drug Discovery and Development Institute (IMS) Strasbourg 67081 France; ^8^ Plateforme GENOMAX Institut thématique interdisciplinaire (ITI) de Médecine de Précision de Strasbourg Transplantex NG Strasbourg 67000 France; ^9^ Laboratoire de Spectrométrie de Masse BioOrganique (LSMBO) IPHC UMR 7178 CNRS Université de Strasbourg Infrastructure nationale ProFI FR 2048 Strasbourg 67037 France; ^10^ Service d'Immunologie Biologique Plateau Technique de Biologie Pôle de Biologie Nouvel Hôpital Civil Hôpitaux Universitaires de Strasbourg 1 Place de l'Hôpital Strasbourg 67091 France; ^11^ Institut Pluridisciplinaire Hubert Curien CNRS UMR 7178 Strasbourg 67037 France; ^12^ Institut Lumière Matière Université Claude Bernard – Lyon 1 CNRS UMR 5306 Villeurbanne 69622 France; ^13^ Institut Universitaire de France (IUF) Paris 75231 France

**Keywords:** immunotherapy, nanomaterial, proteogenomics

## Abstract

Targeting the immune system with nanoparticles (NPs) to deliver immunomodulatory molecules emerged as a solution to address intra‐tumoral immunosuppression and enhance therapeutic response. While the potential of nanoimmunotherapies in reactivating immune cells has been evaluated in several preclinical studies, the impact of drug‐free nanomaterials on the immune system remains unknown. Here, the molecular and functional response of human NK cells and pan T cells to a selection of five NPs that are commonly used in biomedical applications are characterized. After a pre‐screen to evaluate the toxicity of these nanomaterials on immune cells, ultrasmall silica‐based gadolinium (Si‐Gd) NPs and poly(lactic‐*co*‐glycolic acid) (PLGA) NPs are selected for further investigation. Bulk RNA‐sequencing and flow cytometry analysis showcase that PLGA NPs trigger a transcriptional priming toward activation in NK and pan T cells. While PLGA NPs improved NK cells anti‐tumoral functions in a cytokines‐deprived environment, Si‐Gd NPs significantly impaired T cells activation as well as functional responses to a polyclonal antigenic stimulation. Altogether, PLGA NPs are identified as an attractive strategy for reactivating the immune system of cancer patients.

## Graphical abstract

Nanoparticles are used to improve immunomodulatory therapies, but the impact of drug‐free nanomaterials on the immune system remains unknown. Screening common biomedical nanoparticles (NPs) reveals that PLGA NPs enhanced NK cell anti‐tumoral activity and transcriptional activation in human NK and pan T cells. In contrast, Si‐Gd NPs impaired T cell activation and functional responses. These results suggest that PLGA NPs could boost immune responses in cancer patients.

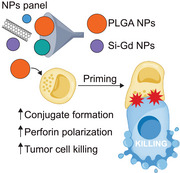

## 1 Introduction

Over the past decade, immunotherapies have become increasingly attractive for cancer treatment.^[^ ^1^, ^2^, ^3^, ^4^ ^]^ Among them, adoptive cell therapy (ACT) emerges as one of the most promising approaches.^[^ ^5^ ^]^ These therapies aim to modify ex vivo the immune cells to induce the expression of an exogenous antigen receptor, enabling the detection of tumor antigens and hence resulting in potent anti‐tumoral responses. T cells isolated from blood or tumor tissue were the first genetically reprogrammed immune cells for ACT.^[^ ^6^, ^7^, ^8^ ^]^ The infusion of autologous modified T cells demonstrates positive clinical outcomes in relapsed hematological malignancies,^[^ ^9^, ^10^ ^]^ yet, adoptively transferred T cells are often associated with severe life‐threatening side effects such as graft‐versus‐host disease, cytokine release syndrome, and neurological toxicities.^[^ ^11^ ^]^ To overcome these limitations, emerging strategies utilizing the cytotoxic activity of non‐MHC restricted NK cells are being evaluated clinically.^[^ ^12^ ^]^ Allogeneic engineered NK cells administered to patients have shown tremendous potential to treat cancer patients, as they do not induce the abovementioned limitations while offering the possibility to be used off‐the shelf.^[^ ^13^, ^14^ ^]^ In contrast to hematological malignancies, intratumoral infiltration of immune cells is restricted in solid tumors that quickly build an immunosuppressive tumor microenvironment (TME), which induces a rapid functional impairment of immune cells.^[^ ^15^, ^16^, ^17^, ^18^, ^19^ ^]^ The emergence of cancer immunotherapy approaches allows for tackling the TME immunosuppression, but these therapies are associated with a low response rate and life‐threatening immune‐related adverse events.^[^ ^20^, ^21^, ^22^, ^23^ ^]^ Consequently, improving cancer immunotherapy efficacy and safety remains a priority.

One of the most promising solutions lies in the utilization of nanoimmunotherapy approaches to specifically target immune cells and to promote immune cell‐mediated anti‐tumoral responses through controlled spatiotemporal immunomodulation.^[^ ^24^ ^]^ Specifically, nanoimmunotherapies have been evaluated using carbon nanotubes (CNTs) or a combination of CNTs and poly(lactic‐*co*‐glycolic acid) (PLGA) polymeric nanoparticles (NPs) conjugated with peptide‐loaded MHC‐I, and the co‐stimulatory ligand anti‐CD28 to act as artificial antigen‐presenting cells, which are pivotal for ex vivo T cell expansion in ACT manufacturing processes.^[^ ^25^, ^26^ ^]^ In addition, polymeric NPs have also been used to deliver immune‐reactivating agents (such as the TGFβR1 inhibitor SD‐208) directly to tumor‐infiltrating T cells in vivo.^[^ ^27^ ^]^ This class of NPs has also enabled the generation of genetically engineered immune cells through ex vivo and in vivo delivery of CAR‐encoding plasmids, providing a virus‐free alternative for transduction.^[^ ^28^, ^29^ ^]^ More recently, ultrasmall metallic NPs decorated with anti‐PD‐L1 V_H_H were developed to monitor in vivo the expression of this immune checkpoint molecule in cancer.^[^ ^30^ ^]^ While these nanoimmunotherapies show promising results in preclinical trials, most reported nanotoxicology evaluations have focused on cargo‐loaded NPs on primary murine cells or human immortalized cell lines.^[^ ^31^, ^32^ ^]^ In contrast, the investigation of NPs' chemistry impact on primary human immune cells at subcellular levels remains limited. Capturing these material‐specific effects on human immune cells is crucial for developing and implementing NPs in relevant clinical settings.^[^ ^33^ ^]^

Here, we use drug‐free NPs to precisely investigate the impact of their material on innate NK cells and adaptive pan T cells sourced from healthy donors to explore the fate of NPs post‐internalization in immune cells, to ensure their safety profile, and guide the selection of specific NPs based on the therapeutic need. To conduct pre‐screening studies integrating cytotoxicity assessments and comprehensive proteomic analysis, we selected five NPs from the broad array being explored in both pre‐clinical and clinical research. Hence, we prioritized a rational selection of nanomaterials that are representative of lipidic, organic, and inorganic nanomaterial classes, ensuring that key variables such as material composition, size, and functionalization could be effectively evaluated. The nanomaterials included in this study were chosen not only based on their clinical relevance and frequent use in biomedical applications but also to address specific questions regarding their structure‐activity relationships. Hence, we evaluated ultrasmall polysiloxane‐based NPs chelated with gadolinium (Si‐Gd NPs) or terbium (Si‐Tb NPs) to explore the role of metal chelation and core composition in modulating immune responses. Additionally, we investigated the impact of NP size by comparing these ultrasmall Si‐Tb NPs to larger terbium fluoride nanoparticles (Tb NPs). Furthermore, we incorporated lipid‐polymer hybrid PLGA NPs and carbon nanotubes (oxCNTs) to examine how diverse physicochemical properties influence cytotoxicity and immune activation. This strategic approach enabled us to balance a broad characterization of nanomaterials with a focused comparative assessment of critical material attributes. By selecting complementary systems, we provide novel insights into how key properties dictate immune cell interactions and highlight generalizable trends that are necessary for the design of future nanoimmunotherapeutics.

Based on these results, we selected two NPs with minimal cytotoxicity and effects on the proteome. We further evaluated their internalization in primary immune cells and highlighted efficient internalization of the two NPs in both primary NK and pan T cells, with CD4^+^ T cells displaying higher internalization potential when compared to CD8^+^ T cells. Proteogenomic mapping of immune cells response to the selected nanomaterial revealed that PLGA NPs trigger pro‐inflammatory transcriptional programs in NK and pan T cells, while Si‐Gd NPs do not alter their transcriptomes. We further probed the effect of NPs on the functional behavior of immune cells and observed that PLGA NPs transcriptional priming of NK cells improved the anti‐tumoral function of NK cells. Conversely, Si‐Gd NPs treatment impaired the pan T cells responses to polyclonal antigenic stimulation. This study provides a unique resource of the impact of NP materials on human NK and pan T cells that will be beneficial to the tailoring of nanoimmunotherapeutic strategies in oncology.

## 2 Results and Discussion

### 2.1 Unequal Internalization and Subsequent Toxicities of Nanomaterials on Human Innate and Adaptative Lymphocytes

**Figure 1:**
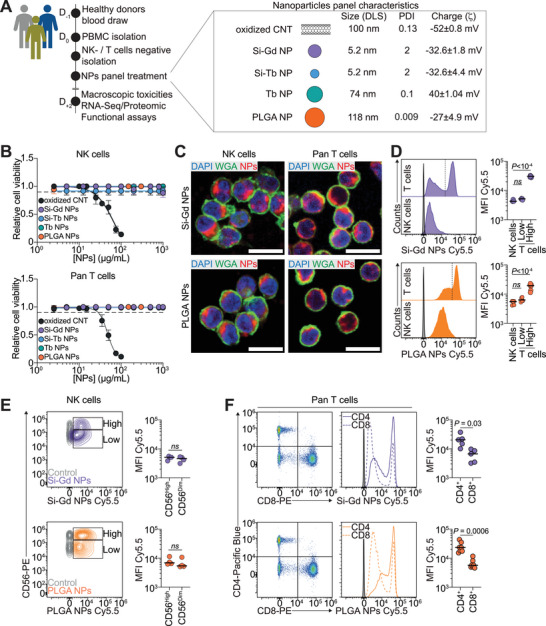
Nanomaterials internalization varies across human NK cells and pan T cells. A) Infographics illustrating the pipeline of the assessment of nanomaterial impact on primary human NK‐ and pan T cells. Nanomaterial physicochemical characteristics are displayed on the right. B) Nanomaterials impact on NK cells (upper panel) and pan T cells (lower panel) viability measured using CellTiterGlo assay. Data are representative of 3 independent donors. Data are presented as mean ± s.d. C) Representative confocal micrographs of NK cells and pan T cells showing internalized Si‐Gd NPs and PLGA NPs after 4 h co‐incubation at 37 °C. In green Wheat Germ Agglutinin (WGA), in red nanomaterial (Cyanine5.5‐labelled), in blue nuclei (DAPI). Scale bar = 10 µm. D) Flow‐cytometry assessment of nanomaterials internalization in NK‐ and pan T cells after 48 h co‐incubation at 37 °C. Left: Representative histograms of the relative internalization of Si‐Gd NPs (upper panel) and PLGA NPs (lower panel). Grey histogram: untreated control. Right: Quantification of the mean fluorescent intensity signal of the Cyanine5.5‐labelled nanomaterials. Data are representative of 3 to 6 independent donors and analyzed by a One‐way ANOVA test with the original FDR method of Benjamini‐Hochberg after assessment of their Gaussian distribution by Shapiro‐Wilk test. E) Flow‐cytometry assessment of nanomaterials internalization in CD56^High^ and CD56^Low^ NK cells after 48 h co‐incubation at 37 °C. Left: Representative flow cytometry contour plots of the relative internalization of Si‐Gd NPs (upper panel) and PLGA NPs (lower panel). Right: Quantification of the mean fluorescent intensity signal of the Cyanine5.5‐labelled nanomaterials. Data are representative of 4 independent donors and analyzed by a Student's t‐test (Si‐Gd NPs) or Mann‐Whitney test (PLGA NPs) after assessment of their gaussian distribution by Shapiro‐Wilk test. F) Flow‐cytometry assessment of nanomaterials internalization in CD4^+^ and CD8^+^ pan T cells after 48 h co‐incubation at 37 °C. Left: Representative flow cytometry contour plots of the relative internalization of Si‐Gd NPs (upper panel) and PLGA NPs (lower panel). Grey histogram: untreated control. Right: Quantification of the mean fluorescent intensity signal of the Cyanine5.5‐labelled nanomaterials. Data are representative of five to seven independent donors and analyzed by a Student's t‐test with Welch's correction (Si‐Gd NPs) or Mann‐Whitney test (PLGA NPs) after assessment of their Gaussian distribution by the Shapiro‐Wilk test.

We use a library of five NPs, composed of i) oxidized carbon nanotubes (oxCNTs), ii) ultrasmall polysiloxane‐based gadolinium (Si‐Gd) NPs and terbium (Si‐Tb) synthesized with a bottom‐up one‐pot synthesis,^[^ ^34^ ^]^ as well as iv) unfunctionalized terbium fluoride (Tb) NPs and v) PLGA NPs (**Figure** **1**A). These five NPs were specifically chosen to enable direct comparisons between representative examples of pre‐clinically and clinically relevant^[^ ^35^ ^]^ inorganic (Si‐Gd, Si‐Tb, Tb NPs), polymeric (PLGA NPs), and carbon‐based (oxCNTs) materials, allowing us to systematically assess the impact of diverse physicochemical parameters covering a large range of hydrodynamic diameter distributions (from 5 to 120 nm) and ζ‐potentials (from −50 to 40 mV), representing a wide spectrum of the nanomedicine field (Figure S1, Supporting Information) on human immune cells. PLGA NPs were selected as the representative polymeric platform due to their FDA‐approved status and distinct advantages for immunological studies, including superior stability in biological fluids, tunable degradation kinetics enabling controlled release, and inherent immunomodulatory properties through complement activation, features that distinguish them from lipid‐based systems. While liposomes and lipid NPs have clinical relevance, their lipid membrane fusion mechanisms represent fundamentally different cellular interaction profiles compared to the sustained‐release polymeric degradation of PLGA, making PLGA more representative of the broader polymeric nanomaterial class. The inorganic Si‐Gd, Si‐Tb were included to represent theranostic applications, while the systematic size and composition variations (ultrasmall Si‐Tb versus larger Tb NPs) enable assessment of structure‐activity relationships. oxCNTs were incorporated to capture the unique immunological effects of 1D morphology not present in spherical particles. This selection strategy ensures comprehensive coverage of major nanomaterial categories while maintaining clinical relevance and avoiding mechanistically redundant systems. To assess the impact of these NPs on primary human immune cells, we negatively isolate NK and pan T cells from healthy donor buffy coats with high purity (>95%) and subject them to a cytotoxicity‐driven screening test (Figure S3A,B, Supporting Information). We incubate the sorted NK and pan T cells with increasing NPs concentration for 48 h (Figure 1B). We observe that oxCNTs markedly decrease NK cells (IC_50_ = 68.37 µg mL^−1^) and pan T cells (IC_50_ = 47.65 µg mL^−1^) viability at low concentrations. Inversely, the other tested nanomaterials do not significantly decrease immune cells (Figure 1B). We perform a whole proteome (liquid chromatography‐tandem mass spectrometry, LC‐MS/MS) analysis to further assess nanomaterials impact on immune cells (Figure S4A, Supporting Information).

While terbium‐based NPs (Si‐Tb and Tb NPs) do not overtly affect immune cell viability (Figure 1B), the whole proteome analysis reveals an upregulation of proteins linked with necroptosis (Figure S4A, Supporting Information). Based on this initial pre‐screening that combines proteomic and viability read‐outs, we exclude cytotoxic oxCNT and terbium‐based NPs as they appear unattractive for further preclinical development in this context. On the other hand, Si‐Gd and PLGA NPs display no toxicity towards primary human immune cells (IC_50_ > 1 mg mL^−1^) (Figure 1B) and the observed deregulated proteins are not associated with specific gene ontology terms (Figure S4A, Supporting Information). We thus decided to further evaluate their impact on immune cells using concentrations of 150 µg mL^−1^ of Si‐Gd NPs^[^ ^36^ ^]^ and 50 µg mL^−1^ of PLGA NPs^[^ ^37^ ^]^ (median of the tested range for the immune cells viability assay).

We next confirm that Cyanine5.5‐labelled Si‐Gd NPs and PLGA NPs efficiently penetrate NK and pan T cells despite their low phagocytic potential (Figure 1C). Through an incubation on ice, we highlight that Cyanine5.5‐labelled Si‐Gd NPs and PLGA NPs internalization in NK and pan T cells rely on their metabolism (Figure S3B, Supporting Information). Furthermore, we assess NPs cellular uptake via flow cytometry 48 h post‐treatment. We observe that Si‐Gd NPs and PLGA NPs are uniformly internalized in NK cells, forming a single continuous population with no significant differences between CD56^High^ and CD56^Low^ NK cells (Figure 1D–E). Conversely, we notice that pan T cells exhibited two distinct populations based on the uptake levels –, i.e., one with low internalization rates comparable to NK cells and another with significantly higher levels of both NPs (Figure 1D). Further flow cytometric analysis revealed that the high internalizing T cell population predominantly comprises CD4^+^ T cells, while the low internalizing population is enriched in CD8^+^ T cells (Figure 1F). Although the exact mechanism underlying the difference in internalization abilities between CD4^+^ and CD8^+^ T cells remains unclear, it might originate from CD4^+^ T cells intrinsic higher metabolic potential^[^ ^38^ ^]^ and unconventional phagocytic potential, a feature not observed in CD8^+^ T cells.^[^ ^39^, ^40^ ^]^ Importantly, increasing the concentration of NPs resulted in a dose‐dependent uptake in NK cells and low‐internalizing pan T cells (Figure S4C, Supporting Information). At the opposite, the high internalizing pan T cells population reached a plateau starting at the lowest tested Si‐Gd NPs concentration and only a marginal increase in PLGA NPs uptake at higher doses (Figure S4D, Supporting Information).

Altogether, we demonstrate that Si‐Gd NPs and PLGA NPs are successfully internalized by NK and pan T cells without associated toxicities. Yet, NK cells and CD4^+^ or CD8^+^ T cells are not equal with regards to nanomaterials uptake.

### 2.2 PLGA NPs Trigger a Pro‐Inflammatory Signature in Immune Cells

**Figure 2:**
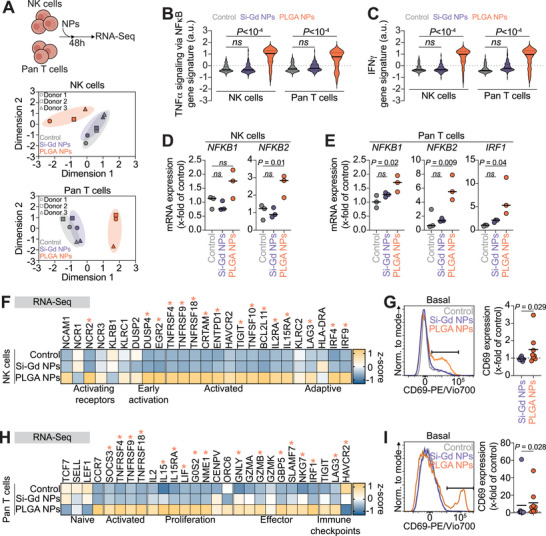
PLGA NPs transcriptionally prime immune cells activation. A) Transcriptomic impact of Si‐Gd NPs and PLGA NPs treatment on immune cells. Upper panel: Schematic representation of the pipeline of the assessment of nanomaterial impact on primary human NK‐ and pan T cells transcriptome. Lower panel: Principal component analysis of the entire transcriptomic profile of NK cells and pan T cells, untreated and treated with Si‐Gd NPs or PLGA NPs. Each point represents one independent replicate. Axis labels represent the percent of variance as in the respective principal components (dimension 1 and dimension 2). B,C) Violin plots comparing aggregate expression distribution of genes related to TNFα signaling via the NFκB pathway (B) or related to IFNγ signaling (C) according to the MSigDB Hallmark 2024 database. The solid line within each violin represents the median, and dotted lines represent quartiles. Data are analyzed by a Kruskal‐Wallis test with original FDR method of Benjamini‐Hochberg after assessment of their gaussian distribution by Shapiro‐Wilk test. D) NK cells expression of *NKFB1* and *NFKB2* mRNA expression as fold change relative to untreated control calculated using the 2^−∆∆Ct^ method (housekeeping: *GAPDH*). Data are representative of three independent donors and analyzed by One‐way ANOVA test with the original FDR method of Benjamini‐Hochberg after assessment of their Gaussian distribution by Shapiro‐Wilk test. **E**. Pan T cells expression of *NKFB1*, *NFKB2* and *IRF1* mRNA expression as fold change relative to untreated control calculated using the 2^−∆∆Ct^ method (housekeeping: *GAPDH*). Data are representative of three independent donors and analyzed by One‐way ANOVA test with the original FDR method of Benjamini‐Hochberg after assessment of their Gaussian distribution by the Shapiro‐Wilk test. **F**. Heat‐map showing Z‐score values for RNA expression of NK cells activation and function‐associated genes. Gene names are represented on the top, while associations are represented on the bottom. Stars represent significant statistical difference between untreated control NK cells and PLGA NPs‐treated NK cells. G) Flow‐cytometry analysis of NK cells activation. Left: Representative histograms of CD69 expression at the NK cells surface in the different treatment conditions. Right: Quantification of CD69 expression as fold change relative to untreated control. Data are representative of seven to eight independent donors and analyzed by a Mann‐Whitney test after assessment of their Gaussian distribution by the Shapiro‐Wilk test. H) Heat‐map showing Z‐score values for RNA expression of pan T cells activation and function‐associated genes. Gene names are represented on the top, while associations are represented on the bottom. Stars represent a significant statistical difference between untreated control pan T cells and PLGA NPs‐treated pan T cells. G) Flow‐cytometry analysis of pan T cells activation. Left: Representative histograms of CD69 expression at the pan T cells surface in the different treatment conditions. Right: Quantification of CD69 expression as fold change relative to untreated control. Data are representative of eight independent donors and analyzed by a Mann‐Whitney test after assessment of their Gaussian distribution by the Shapiro‐Wilk test.

To provide a refined analysis of the impact of the NPs on human NK and pan T cells, we perform a gene expression analysis using bulk RNA sequencing after 48 h of treatment. Unsupervised analysis of the entire transcriptome using principal component analysis (PCA) reveals that PLGA NPs‐treated populations clustered separately from control (untreated) and Si‐Gd NPs‐treated cells in the principal component space, indicating significant transcriptomic differences (**Figure** **2**A). We identify 249 differentially expressed genes (DEGs) in NK cells and 395 DEGs in pan T cells following PLGA NPs exposure (adjusted *p*‐value ≤ 0.05 and fold‐change > 2) (Figure S5A and Table S1,S2, Supporting Information). In contrast and in agreement with the PCA analysis, only 16 and 11 genes are differentially expressed in Si‐Gd NPs‐treated NK cells and pan T cells, respectively (Figure S5A and Table S1,S2, Supporting Information).

Upstream regulators analysis of the deregulated genes following PLGA NPs treatment reveals that TNF was the most significant activated regulator (NK cells Z‐score 7.79; pan T cells Z‐score 5.354 and *p*‐values of overlap were 2.82 × 10^−42^ and 4.60 × 10^−38^, respectively). Other regulators in the top five included IL1B, NFκB, and IFN, all of which were predicted to be activated upon PLGA NPs treatment (Figure S5B, Supporting Information). When we compared the expected protein‐protein interactions network among upregulated genes in PLGA NPs‐treated pan T cells, we found a complex interaction between TNF, NFκB, and IFN in NK‐ (Figure S5C, Supporting Information) and pan T cells (Figure S5D, Supporting Information).

Using an aggregate expression analysis, we confirm the global upregulation of tumor necrosis factor‐α (TNFα) signaling via the nuclear factor‐κB (NFκB) and interferon gamma (IFNγ) pathways in PLGA NPs‐treated NK cells compared with the untreated and Si‐Gd NPs‐treated populations (Figure 2B,C). Overexpression of key downstream regulators NFκB and IRF1 mRNA is further confirmed by reverse transcription quantitative polymerase chain reaction (RT–qPCR) (Figure 2D,E). In addition to significant enrichments of upregulated genes in GO terms/pathways associated with cytokines signaling and several immune‐associated pathways, we observe that PLGA NPs treatment also induce an upregulation of immune cells specific transcriptional programs such as NK cells cytotoxicity against tumor cells, α/β T cells activation and T_H_1 immune response (Figure S5E, Supporting Information).

When probing transcriptional programs specific to immune cells activation, we observe that PLGA NPs treatment leads to a significant overexpression of genes linked to NK cells activating receptors (*NCR2*/NKp44), immediate‐early response genes (*DUSP4*, and *EGR2*), and co‐stimulatory receptors (*TNFRSF4*/OX‐40, *TNFRSF9*/4‐1BB, *TNFRSF18*/GITR, *CRTAM*, and *TNFSF10*/TRAIL)^[^ ^41^, ^42^ ^]^ (Figure 2F; Table S3, Supporting Information). Correlatively, PLGA NPs‐treatment induce a trend increase in the early activation marker CD69 expression at the basal level in NK cells, which we further confirm by flow cytometry (Figure 2G). PLGA NPs also induced an overexpression of the high‐affinity interleukin‐2 receptor (*IL2RA*/CD25) and NME1 (Fold Change: 2.46; *q*‐value = 4.02 × 10^−4^), which are critical for NK cells activation and proliferation^[^ ^43^ ^]^ (Table S3, Supporting Information). Additionally, PLGA NPs‐treated NK cells displayed an upregulation of *IFNG* (Fold Change: 7.46; *q*‐value = 4.67 × 10^−5^), *ICAM1* (Fold Change: 2.29; *q*‐value = 2.86 × 10^−5^) transcripts required for NK cells killing of tumor cells^[^ ^13^, ^44^ ^]^ as well as *XCL1* (Fold Change: 2.15; *q*‐value = 2.10 × 10^−6^) transcripts linked to cDC1 recruitment and orchestration of adaptive immunity by NK cells^[^ ^45^ ^]^ (Table S3, Supporting Information). Transcriptional programs analysis also revealed that PLGA NPs treatment induced the upregulation of genes associated with an NK cells adaptative/memory phenotype (*LAG3*, *IRF4* and *BCL2L11*/BIM)^[^ ^46^, ^47^, ^48^ ^]^ (Figure 2F; Table S3, Supporting Information). These NK cells also overexpressed *CCR7* (Fold Change: 2.97; *q*‐value = 8.63 × 10^−10^) and *CD83* (Fold Change: 3.38; *q*‐value = 1.07 × 10^−23^), genes associated with migratory and helper phenotypes leading to high ability to produce IFN‐γ .^[^ ^49^ ^]^

Similarly, transcriptional programs analysis of PLGA NPs treated pan T cells present a positive z‐score for genes related with activation (*TNFRSF4*/OX‐40, *TNFRSF9*/4‐1BB, *TNFRSF18*/GITR), proliferation (*IL15*/*IL15RA, LIF*), and cell division (*G0S2*, *NME1*)^[^ ^50^, ^51^ ^]^ as well as effector functions (*GNLY*, *GZMB*, *SLAMF7*, *NKG7*)^[^ ^52^, ^53^ ^]^ (Figure 2H; Table S3, Supporting Information). Alongside, PLGA NPs‐treated pan T cells displayed an upregulation of ATF‐like transcription factors such as *BATF* (Fold Change: 2.38; *q*‐value = 0.001) and *BATF3* (Fold Change: 2.34; *q*‐value = 0.04), which are essential checkpoints for early effector T cells differentiation.^[^ ^54^, ^55^ ^]^ Furthermore, as highlighted in the differentially expressed term analysis (Figure S5C, Supporting Information), these effector‐primed T cells displayed a T_H_1 transcriptional landscape (*STAT1*, *ADAM12*)^[^ ^56^, ^57^ ^]^ as well as a reduction in T_H_2 transcription factors transcripts (*GATA3*) and cytokines (*IL12B*, *TNF*, *IL17F*, *XCL1, CCL3L1*, *CCL4L1*) known to prime and to be expressed during T_H_1/TH_17_‐like pro‐inflammatory response^[^ ^58^, ^59^, ^60^ ^]^ (Table S3, Supporting Information). Similarly to NK cells and in accordance with the upregulation of activation/effector‐associated genes, PLGA NPs treatment increased CD69 expression at the basal level in pan T cells as observed by flow cytometry (Figure 2I). We confirmed such priming with a nanomaterial‐induced dose‐dependent decrease in CD3 expression (Figure S6A,B, Supporting Information) and with an increase in pan T cells size (Figure S6C–D, Supporting Information), which are classically associated with their activation.^[^ ^61^ ^]^ On the other hand, and as expected, Si‐Gd NPs did not induce pan T cells basal activation (Figure S6A–E, Supporting Information). Besides, transcriptional activation of pan T cells by PLGA NPs was further underpinned by the induction of multiple gene families associated with canonical IFN response module (*IFIT1*, *IFIT2*, *IFIT3*, *IFITM1*, *IFITM2*, *IRF1*, *IFI44*, *IFI44L*, *STAT1*, *GBP1*, *GBP4*, *GBP5*)^[^ ^52^, ^62^, ^63^ ^]^ (Table S2, Supporting Information). Of note, we also found a significant increase in the expression of immune checkpoint transcripts (*LAG3*, *HAVCR2*/TIM3) (Figure 2H; Table S3, Supporting Information) that can be counteract by the upregulation of *EGR2* (Table S3, Supporting Information) whose role was recently described in maintaining anti‐tumor responses of exhausted T cells.^[^ ^64^, ^65^ ^]^

Altogether, we identify a transcriptional priming in NK and pan T cells upon drug‐free PLGA NPs treatment, but not with Si‐Gd NPs, through bulk RNA sequencing of immune cells. Flow cytometry analyses further exhibit that drug‐free PLGA NPs exposure was sufficient to trigger NK and pan T cells activation.

### 2.3 PLGA NPs Enhance Anti‐Tumor Functions of NK Cells

**Figure 3:**
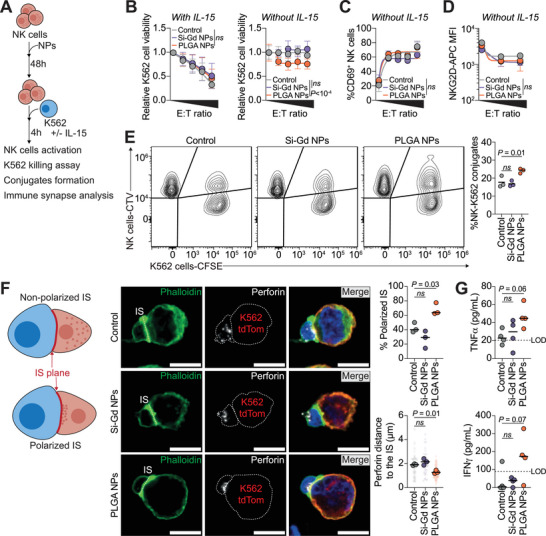
PLGA NPs enhance anti‐tumor functions of NK cells by promoting perforin polarization at the immune synapse. A) Schematic representation of the pipeline of the assessment of Si‐Gd NPs and PLGA NPs functional impact on primary human NK cells. B) Flow‐cytometry assessment of K562 lysis induced by increasing nanomaterials‐treated NK cells ratio (0.625:1; 1.25:1; 2.5:1, and 5:1) after 4 h of co‐incubation with 10 ng/mL of IL‐15 (left) or without (right). Data are representative of six to eight independent donors and analyzed by a Two‐way ANOVA corrected with the original FDR method of Benjamini‐Hochberg. C) Percentage of CD69‐positive NK cells assessed after co‐incubation with K562 cells for 4 h. Data are representative of 3 independent donors and analyzed by a Two‐way ANOVA corrected with the original FDR method of Benjamini‐Hochberg. D) Mean fluorescence intensity of NKG2D expressed at the NK cells surface after co‐incubation with K562 cells for 4 h. Data are representative of three independent donors and analyzed by a two‐way ANOVA corrected with the original FDR method of Benjamini‐Hochberg. E) Representative conjugate formation assay. Left: Representative contour plots conjugates formed between CTV‐stained NK cells and CFSE‐stained K562 cells after co‐incubation at a 1:1 ratio for 20 min. Right: Quantification of the percentage of conjugates in each condition. Data are representative of three independent donors and analyzed by a two‐way ANOVA corrected with the original FDR method of Benjamini‐Hochberg. F) Nanomaterials‐treated NK cells immune synapse (IS) formation with K562. Left: Schematic representation of IS polarization and perforin distance evaluation. Center: Representative confocal micrographs of NK‐K562 IS. In green Phalloidin‐iFluor488, in white perforin dG9 (Alexa Fluor 647), in red K562 palmitoylated‐tdTomato and in blue nuclei (DAPI). Scale bar = 10 µm. Right: Percentage of polarized IS (upper panel) and mean perforin distance to the IS (lower panel). Data are representative of between 43 and 73 synapses coming from three independent donors displayed as mean per experiment. Data are analyzed by a One‐way ANOVA test with the original FDR method of Benjamini‐Hochberg after assessment of their Gaussian distribution by the Shapiro‐Wilk test. **G**. Concentration of TNFα (upper panel) and IFNγ (lower panel) released in the supernatant after 4 h of NK cells co‐incubation with K562 cells at a 2:1 ratio. Data are representative of four independent donors and analyzed by a One‐way ANOVA test with the original FDR method of Benjamini‐Hochberg (TNFα) or by a Kruskal‐Wallis test (IFNγ) after assessment of their Gaussian distribution by the Shapiro‐Wilk test. LOD: limit of detection.

As PLGA NPs could transcriptionally prime NK cells, we sought to investigate whether they could functionally activate their tumor‐lytic properties (**Figure** **3**A; Figure S7A,B, Supporting Information). We co‐cultured NPs‐treated NK cells with their prototypical targets lacking MHC class I molecules, the K562 cells, over 4 h with or without supplementing the pro‐survival cytokine IL‐15 to mimic tumor microenvironments that often lack pro‐survival cytokines required by cytotoxic NK cells^[^ ^66^ ^]^ (Figure 3A). With IL‐15, both Si‐Gd NPs and PLGA NPs‐treated NK cells successfully clear the K562 targets (Figure 3B). Without supplementary IL‐15, only PLGA NPs‐treated NK cells efficiently induce killing of 25% of the target cells (Figure 3B). Of note, NK cells anti‐tumoral functions alterations observed in control NK cells or upon Si‐Gd NPs treatment are not resulting from an impaired NK cell activation, as all conditions exhibited similar CD69 expression levels (Figure 3C). In addition, both NPs‐treatment do not impair K562 target cells recognition, as observed through the measurement of available NKG2D receptor at the NK cells surface by flow cytometry (Figure 3D). Nevertheless, without IL‐15, PLGA NPs treatment induces a significant increase in the number of conjugates between NK cells and their K562 target cells, as measured by flow cytometry (Figure 3E). Since efficient cytotoxic granule polarization at the immune synapse (IS) is also critical for the killing capacity of NK cells, we next induced IS formation and measured the distance between mature perforin and the plasma membrane of NK‐K562 synapses (Figure 3F), using stable expression of palmitoylated tdTomato as a K562 membrane marker (Figure S6C, Supporting Information). Without IL‐15, PLGA NPs treatment favors cytotoxic granules convergence at the IS, as measured by the reduction of granules distance to the IS (Figure 3F). Concomitantly, PLGA NPs treatment increases the percentage of NK cells presenting a polarized IS (Figure 3F). In contrast, neither Si‐Gd NPs treated nor untreated NK cells present polarized IS (or reduced distance between perforin and the IS) (Figure 3F), suggesting that, as expected, PLGA NPs treatment induces efficient K562 cells lysis through an increase in NK cells perforin polarization. Coming back to our proteogenomic analysis, we highlight that PLGA NPs induce the upregulation of proteins associated with NK cells maturation (IKZF3),^[^ ^67^ ^]^ cell adhesion (VLA‐1: ITGA1/ITGB1),^[^ ^68^ ^]^ and vesicle transport (STX11)^[^ ^69^ ^]^ (Figure S7D, Supporting Information). Conversely, Si‐Gd NPs repress the expression of these proteins and stimulate the expression of proteins associated with NK cells inhibitory signaling (HLA‐E and GSK3B)^[^ ^70^, ^71^, ^72^ ^]^ that are less or not expressed in PLGA NPs‐treated NK cells (Figure S7D, Supporting Information). NK cell‐induced cytotoxicity requires both polarization of the cytotoxic granules and their fusion with the plasma membrane, allowing the release of their cytotoxic content. We next monitor NK cells degranulation by dosing the cytotoxic cytokines TNFα^[^ ^73^ ^]^ and IFNγ ^[^ ^74^ ^]^ in the supernatant after 4 h of NK cells co‐incubated with their target K562 cells. Increased cytotoxic granules polarization in PLGA NPs‐treated NK cells is corroborated by a trend, yet non‐significant, augmentation in TNF‐α and IFN‐γ concentrations when compared to control and Si‐Gd NPs‐treated NK cells (Figure 3G).

According to these results, we demonstrate that NK cells transcriptional priming towards an activated phenotype induced by PLGA NPs results in an enhancement of their anti‐tumoral cytotoxic capacity in vitro in a cytokines‐deprived (i.e., immunosuppressive) environment.

### 2.4 Silica‐Based Gadolinium NPs Treatment Impairs Pan T Cells Functions

**Figure 4:**
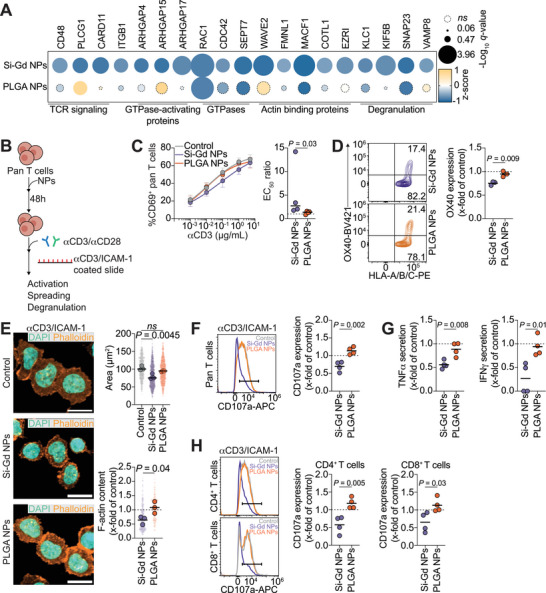
Silica‐based gadolinium NPs (Si‐Gd NPs) treatment impairs pan T cells spreading and degranulation. A) Bubble plot showing Z‐score values for protein expression associated with pan T cells functions. Protein names are represented on the top, while associations are represented on the bottom. Bubble size represents ‐Log_10_(*q*‐value). Dashed lines and bubbles correspond to non‐significantly deregulated proteins. B) Schematic representation of the pipeline of the assessment of Si‐Gd NPs and PLGA NPs functional impact on primary human pan T cells. C) Pan T cells sensitivity to polyclonal activation. Left: Percentage of CD69‐positive pan T cells assessed after culture on a culture plate coated with increasing concentration of anti‐CD3 antibodies for 24 h. Right: Half maximal effective concentration (EC_50_) calculated from the dose‐response curve in the left graph. Data are representative of four independent donors and analyzed by a Mann‐Whitney test after assessment of their Gaussian distribution by the Shapiro‐Wilk test. D) Flow‐cytometry assessment of OX‐40 expression at the pan T cells surface after activation with 5 µg mL^−1^ of anti‐CD3 and 5 µg mL^−1^ of anti‐CD28 antibodies for 24 h. Left: Representative flow cytometry contour plots of the relative OX‐40 expression in activated pan T cells previously treated for 48 h with Si‐Gd NPs (upper panel) or PLGA NPs (lower panel). Right: Quantification of OX‐40 expression as fold change relative to untreated control. Data are representative of three independent donors and analyzed by a Student's t‐test after assessment of their Gaussian distribution by the Shapiro‐Wilk test. E) Nanomaterials‐treated pan T cells spreading on an antigen‐presenting cells mimicking surface. Left: Representative confocal micrographs of pan T cells spreading. In orange Phalloidin‐iFluor488, in cyan nuclei (DAPI). Scale bar = 10 µm. Right: Pan T cells median spreading area. Left: Relative filamentous actin content in pan T cells as fold change relative to untreated control. Data are representative of 3 independent donors and analyzed by a One‐way ANOVA test with the original FDR method of Benjamini‐Hochberg (Spreading area) or a Student's t‐test (F‐actin content) after assessment of their gaussian distribution by Shapiro‐Wilk test. F) Flow‐cytometry analysis of pan T cells degranulation. Left: Representative histograms of CD107a (LAMP‐1) expression at the pan T cells surface after activation with 5 µg/mL of anti‐CD3 and 5 µg/mL of anti‐CD28 antibodies for 4 h. Right: Quantification of CD107a expression as fold change relative to untreated control. Data are representative of four independent donors and analyzed by a Student's t‐test after assessment of their Gaussian distribution by the Shapiro‐Wilk test. G) TNFα (left panel) and IFNγ (right panel) secretion in the supernatant after activation with 5 µg mL^−1^ of anti‐CD3 and 5 µg mL^−1^ of anti‐CD28 antibodies for 4 h. Data are represented as fold change relative to untreated control. Data are representative of four independent donors and analyzed by a Student's t‐test after assessment of their Gaussian distribution by the Shapiro‐Wilk test. **H**. Flow‐cytometry analysis of CD4^+^ and CD8^+^ T cells degranulation. Left: Representative histograms of CD107a (LAMP‐1) expression at the CD4^+^ T cells (upper panel) and CD8^+^ T cells (lower panel) surface after activation with 5 µg/mL of anti‐CD3 and 5 µg mL^−1^ of anti‐CD28 antibodies for 4 h. Right: Quantification of CD107a expression as fold change relative to untreated control. Data are representative of four independent donors and analyzed by a Student's t‐test after assessment of their Gaussian distribution by the Shapiro‐Wilk test.

When investigating the impact of NPs on T cell functions, we notice from the unbiased proteomic approach previously described (Figure S4A, Supporting Information) that proteins involved in TCR downstream signaling are significantly downregulated (CD48, PLCG1, and CARD11)^[^ ^75^, ^76^, ^77^ ^]^ upon Si‐Gd NPs treatment (**Figure** **4**A). These modifications are accompanied by a significant reduction in proteins associated with pan T cells cytoskeletal remodeling, including small GTPase (CDC42, RAC1 or SEPT7),^[^ ^78^, ^79^ ^]^ actin binding proteins (Ezrin, EZRI or WAVE2)^[^ ^80^, ^81^ ^]^ as well as formins (formin‐like‐1, FMNL1)^[^ ^82^ ^]^ (Figure 4A). Si‐Gd NPs‐treated pan T cells also display a significant decrease in the levels of integrin β1, which sustains cytotoxic T cells functions^[^ ^83^, ^84^ ^]^ and their efficient intra‐tumoral infiltration.^[^ ^85^ ^]^ Similarly, we also observe a significant diminution in proteins required for terminal transport (KLC1 and KIF5B)^[^ ^86^ ^]^ and membrane fusion (SNAP23 and VAMP8)^[^ ^87^, ^88^ ^]^ of lytic granule at the immune synapse (Figure 4A). Incidentally, PLGA NPs have no or little effect on these proteins (Figure 4A). Building on these observations, we aim to test the effect of NPs on pan T cells function. We mimic stimulation of the TCR and co‐receptors by coating surfaces with activating antibodies against CD3 (part of the TCR complex) and CD28 (a costimulatory receptor) and measure how T cells activate and spread, as they would over the surfaces of antigen presenting cells (APCs)^[^ ^89^ ^]^ (Figure 4B). We first observe that both Si‐Gd and PLGA NPs treatment do not prevent global pan T cell activation when comparing CD69 expression after 24 h of culture on a surface coated with increasing concentrations of anti‐CD3 and a fixed concentration of anti‐CD28 antibodies (hereafter, anti‐CD3/CD28) (Figure 4C). This result further suggests that transcriptional priming is antigen‐independent and mediated by intracellular cues. However, in line with TCR signaling proteins downregulation observed in our proteomic data, Si‐Gd NPs‐treated pan T cells require higher anti‐CD3/CD28 concentrations to reach similar activation levels as control and PLGA NPs‐treated pan T cells, suggesting a reduced sensitivity to TCR activation (Figure 4C). We further confirm this weakened TCR signaling by measuring OX‐40 expression, a co‐stimulatory molecule selectively induced after TCR stimulation.^[^ ^90^, ^91^ ^]^ When normalizing to untreated pan T cells, we notice that Si‐Gd NPs reduced by 25% OX‐40 expression (Figure 4D).

Next, we sought to analyze the NPs treatment effect on actin remodeling at the IS. We compare pan T cell spreading on glass surfaces coated with anti‐CD3 together with a recombinant ICAM1‐Fc chimeric protein (Figure 4B). We monitor T cells morphology and cytoskeletal remodeling and observe that Si‐Gd NPs, but not PLGA NPs, significantly reduce cell spreading and F‐actin content (Figure 4E). T cell‐mediated functions require not only spreading on the APCs surface but also degranulation. We thus assess the effect of NPs treatments on the degranulation of pan T cells as well as of their CD4^+^ and CD8^+^ subpopulations. To do so, we follow the cell surface expression of CD107a (LAMP‐1) and cytokines release in T cells activated by anti‐CD3/CD28 and ICAM1‐Fc chimeric protein. When compared to control, CD107a expression is significantly decreased by Si‐Gd NPs treatment in pan T cells from all tested donors, hinting at an inhibition of their degranulation (Figure 4F). This phenotype is associated with a significant twofold reduction in TNFα and IFNγ secretion (Figure 4G). We previously observed that CD4^+^ and CD8^+^ T cells internalized differentially the NPs (Figure 1F) and thus wonder whether the uptake level of NPs affects the T cells functionality. Hence, we further evaluate the CD107a expression of CD4^+^ and CD8^+^ T cells within the pan T cells population. Interestingly, CD4^+^ and CD8^+^ T cells exhibited a similar significant twofold diminution in CD107a expression upon Si‐Gd NPs treatment, suggesting that their detrimental effect already occurs at low NP doses (Figure 4H).

Altogether, we highlight that Si‐Gd NPs treatment widely impairs T cells activation, and functional responses to a polyclonal antigenic stimulation.

## 3 Conclusion

Nanoimmunotherapies, combining nanotechnology and immunotherapy, represent a promising therapeutic strategy to improve anti‐tumoral immune response. Understanding how NPs materials impact immune cells post‐internalization is essential to ensure their safety profile and rationally guide the selection of specific NPs based on the therapeutic need.

Here, we present a pioneering analysis of the material impact of untargeted and drug‐free NPs on two human primary immune cell types. Through a pre‐screen combining cytotoxicity measurement and whole proteome analysis of a panel of five different nanomaterials under preclinical and clinical evaluation, we first highlight the toxicity of oxCNTs and terbium‐based NPs (Tb and Si‐Tb NPs) on NK and pan T cells. Overt toxicity at low concentration induced by oxCNTs might be partially explained by the downregulation of a wide range of proteins associated with cellular metabolic processes, as well as ribosome‐associated proteins, as observed by whole proteome analysis (Figure S4A, Supporting Information). Of note, ribosomal associated proteins modulation was previously observed following co‐culture of Jurkat T cells with carbon‐based NPs.^[^ ^92^ ^]^

Based on this pre‐screen, we identify Si‐Gd NPs and PLGA NPs as promising candidates for further preclinical development. We further examine Si‐Gd NPs and PLGA NPs interactions with immune cells using a proteogenomic approach. Bulk RNA‐sequencing reveals that PLGA NPs triggered a strong transcriptional priming toward activation in NK and pan T cells. We additionally validate by flow cytometry that drug‐free PLGA NPs exposure was sufficient to activate NK and pan T cells, further confirming the transcriptional priming. Upstream regulators and aggregate expression analysis reveal that this priming is, in part, mediated by TNF‐α via NFκB and IFN‐γ pathways. Investigation of the expected protein‐protein interactions network among upregulated genes in PLGA NPs‐treated NK‐ and pan T cells (Figure S5C,D, Supporting Information) further underpin the complex interaction between TNF, NFκB, and IFN pathways. Although our study sheds light on which signaling pathway activated by PLGA NPs triggers NK‐ and pan T cells activation, the initial events and targets engaged during the immune cells‐NPs interaction remain to be identified. One could combine modeling of potential interactions between NPs, their protein corona, and immune sensors such as Toll‐Like Receptors or other Pattern Recognition Receptors (PRRs). Additionally, localization and possible colocalization of NPs with PRRs using high‐resolution live‐cell imaging could solve the mechanisms underlying NK and pan T cell activation. More detailed investigations of NK functions upon NPs exposure reveal that PLGA NPs treatment efficiently enhance NK cells tumoricidal activity in cytokines‐deprived environment. In‐depth analysis showcases that the NK cells functional enhancement is mediated by an increase in conjugate formation, a better mature perforin polarization at the IS associated with a surge in cytotoxic cytokines release. Increased perforin polarization might be partially explained by an enhanced NK cells maturation and vesicles transport as observed in our proteogenomic data. In line with our results, another study recently demonstrated that drug‐free polymer micropatches were able to activate murine neutrophils and induce an N1 anti‐tumoral response.^[^ ^93^ ^]^ Yet, our in vitro analysis does not allow to evaluate how NK cells priming integrates into the global adaptive immune system. Since NK cells are key player in the regulation of adaptive immune responses,^[^ ^94^ ^]^ notably by the recruitment dendritic cells via XCL1^[^ ^45^ ^]^ (that is overexpressed by PLGA NPs‐treated NK cells), thorough in vivo evaluation remained to be performed to decipher ex vivo primed NK cells tumoricidal enhancement on tumor burden regression and systemic immune response induction.

In parallel, explorations of pan T functions expose that Si‐Gd NPs treatment widely impaired their activation and functional responses to a polyclonal antigenic stimulation. Detailed analysis reveals that impairment was similar in CD4^+^ and CD8^+^ T cells despite different internalization rates, suggesting that their detrimental effect already occurs at low doses. Si‐Gd NPs are under clinical evaluation as an MRI contrast agent^[^ ^95^ ^]^ and radio‐enhancer^[^ ^96^ ^]^ (NCT04789486). Si‐Gd NPs increase the radiation effects on tumor cells, inducing higher immunogenic cell death levels and higher immune cells recruitment to the tumor bed.^[^ ^97^ ^]^ Therefore, a careful assessment of the impact of Si‐Gd NPs on recruited immune cells is necessary. In addition, Si‐Gd NPs were recently targeted with a programmed death‐ligand 1 (PD‐L1) V_H_H to monitor immune checkpoint molecules expression in vivo through medical imaging.^[^ ^30^ ^]^ However, human effector T cells are also PD‐L1.^[^ ^98^, ^99^ ^]^ These results suggest that Si‐Gd NPs development as medical imaging tracers to assess immune infiltration in tumors and to predict response to immunotherapy treatments should be carefully evaluated. Interestingly, a previous study exhibited that ultrasmall silica NPs were able to induce a dose‐dependent CD4^+^ and CD8^+^ T cells activation.^[^ ^36^ ^]^ These ultrasmall silica NPs possess a hydrodynamic diameter similar to our Si‐Gd NPs, suggesting that metallic loading can impact T cells response. Further investigations in that sense should be performed to evaluate other metals (such as gold or silver NPs). While transcriptomic analysis reveals that PLGA NPs seemed to promote a T_H_1 anti‐tumoral polarization signature, working with healthy pan T cells we were not able to investigate this aspect of the NPs impact. Indeed, healthy pan T cells do not possess a tumor antigen‐specific TCR. Further evaluation working with T cells sourced from cancer patients or TCR‐engineered T cells is required to evaluate the functional impact of the transcriptional T_H_1 polarization.

Our findings highlight the significant potential of drug‐free PLGA NPs to activate human immune cells via the TNF‐α and NFκB pathways. This study lays a strong foundation for future preclinical and clinical research into PLGA NP‐based immunotherapies. Further exploration, including in vivo evaluations and studies involving tumor‐infiltrating lymphocytes and primary tumor cells, may support the validation of such strategies. A first step, assessing the safety and immuno‐modulatory effects of PLGA NPs in cancer‐specific settings using humanized mouse models, would be valuable. However, replicating the human immune system in mice remains challenging, as these models often display defective NK cells homeostasis^[^ ^100^ ^]^ and limited immune functions due to the absence of lymph nodes for effective immune response mounting,^[^ ^101^ ^]^ and they rely on supraphysiological cytokine expression to compensate.^[^ ^102^ ^]^ Additionally, our results offer valuable insights for the rational selection of NP materials in nanoimmunotherapeutic strategies. We identify drug‐free PLGA NPs as promising candidates for targeted approaches aimed at reactivating the immune response in cancer patients. The intrinsic properties of PLGA NPs may also synergize with immuno‐activating agents, providing a complementary mechanism to effectively modulate and enhance immune responses.

## 4 Experimental Section

### Nanoparticles Synthesis

Nanoparticles were synthesized as previously described. Briefly, carbon nanotubes had been shortened under strong acid conditions (H_2_SO_4_/HNO_3_ 3:1) and sonication for 24 h to introduce a high density of carboxylic groups on their tips and sidewalls.^[^ ^103^ ^]^ The ultra‐small metal‐based NPs were composed of a polysiloxane core, and a shell comprising amine function and metal complexed by DOTAGA (in the same amount) and were provided by the Institut Lumière‐Matière of the University of Lyon. Si‐Gd were synthesized by a top‐down process^[^ ^104^ ^]^ and Si‐Tb were synthesized in a one‐pot protocol.^[^ ^34^ ^]^ Lanthanide‐doped La_0.9_Tb_0.1_F_3_ NPs (hereafter, Tb NPs) were synthesized by dropwise addition of 0.9 LaCl_3_ and 0.1 TbCl_3_ to 3 NH_4_F followed by heating at 150 °C for 12 min and purification, as described in.^[^ ^105^ ^]^ Finaly, hybrid PLGA‐lipid NPs were synthetized via self‐assembly of poly(D,L‐lactide‐*co*‐glycolide) acid (30–60 kDa, lactide:glycolide 50:50; Sigma #P2191) and 1,2‐distearoyl‐sn‐glycero‐3‐phosphoethanolamine‐N‐[carboxy(polyethylene glycol)‐2000] (sodium salt) (DSPE‐PEG‐CO₂H; Avanti Polar Lipids #880 135) through a one‐step nanoprecipitation method as described previously in.^[^ ^106^ ^]^ Briefly, PLGA polymer was dissolved with or without 0.2% cyanine 5.5 carboxylic acid (Lumiprobe #17 090; λ_ex_/λ_em_: 684 nm/710 nm) in acetonitrile at a concentration of 5 mg mL^−1^. DSPE‐PEG‐CO₂H, at a weight ratio of 20% relative to PLGA polymer, was dissolved in 10 mL of 4 wt.% ethanol aqueous solution and stirred vigorously at 65 °C. The PLGA solution was then added dropwise into the lipid solution using a syringe pump (0.5 mL h^−1^) under constant stirring. The entire mixture was kept under gentle stirring for 2 h at room temperature under a chemical hood. The remaining organic solvent and unloaded molecules were removed by washing the NP solution with ultrapure water using an Amicon Ultra‐15 centrifugal filter from Millipore, France (cut‐off: 50 kDa; 3 cycles, 3000 *g*, 10 min) via tangential centrifugation. The final NP formulation was reconstituted in 1 mL of ultrapure water.

### Dynamic Light Scattering

Dynamic light scaterring (DLS) measurements were conducted using a nano‐ZS instrument (Malvern). The suspensions of NPs were prepared in a solution of nanopure water (Milli‐Q). DLS measurements were performed in sets of ten acquisitions. The average hydrodynamic diameters of the NPs were determined by analyzing the DLS correlation function through a regularization fitting method.

### Human NK and Pan T Cells Isolation

Peripheral blood mononuclear cells (PBMC) were obtained by density gradient centrifugation (1200 *g*, 20mins) of buffy coats from healthy volunteer blood donors under written informed consent recruited at Établissement Français du Sang Grand‐Est, Strasbourg, France (agreement A122395/2022). Following erythrolysis (MiltenyiBiotec #130‐094‐183), NK cells and pan T cells were isolated from PBMC by negative magnetic cell‐sorting (MiltenyiBiotec #130‐092‐657and #130‐096‐535) following manufacturer instructions. Post‐sort cell purity was then controlled by flow cytometry on a Attune NxT (Invitrogen) flow cytometer using PE anti‐CD56 (1:100, MiltenyiBiotec #130‐113‐312) and FITC anti‐CD3 (1:100, MiltenyiBiotec #130‐126‐882) staining and ranged from 90% to 99.2% (median 96.3%). Gating strategy is shown in Figure S2 (Supporting Information).

NK‐ and pan T cells (10^6^ cells/mL) were cultured for further experiments in R10 medium: RPMI 1640 (Gibco #72 400 054) containing 2 mM Glutamine, 25 mM HEPES, and completed with 10% v/v of (fetal bovine serum) FBS (Gibco), 100 U mL^−1^ penicillin, 100 µg mL^−1^ streptomycin (PanBiotech #P06‐07100), and 50 µM β‐mercaptoethanol (Gibco #11508916).

### Cell Lines and Cell Line Engineering

The human chronic myelogenous leukemia cell line K562 (ATCC CCL‐243) was cultured under standard conditions (37 °C, 5% CO_2_) using complete R10 medium. Cell viability in vitro was assayed before the functional experiment by a Countess three automated cell counter (ThermoFisher).

For immune synapse formation, K562 cells were engineered to express a palmitoylated tdTomato. Briefly, the tdTomatoDNA fragment from the Addgene plasmid tdTomato‐Lifeact‐7 (#54 528) was amplified by PCR to add the palmitoylation sequence of the *GAP43* gene and then cloned in pJET 1.2 vector according to manufacturer's instructions (ThermoFisher #K1231). The generated Mb‐tdTomato fragment was then cloned into a lentivirus pLSFFV‐IRES‐Blasticidin vector. Lentivirus generated from the pLSFFV‐Mb‐tdTomato‐IRES‐Blasticidin construct was produced by transfection together with 3 additional vectors (pLP1, pLP2, and pLP3‐VSV plasmids) in HEK293T cells using JetPRIME transfection reagent (Polyplus).

For K562 cells transduction, six wells plate was coated with Retronectin (Takara, 10 µg cm^−2^) for 2 h at room temperature. Wells were washed in PBS before blocking with 2% BSA in PBS for 30 min. After a wash with PBS, 400.000 K562 cells were seeded and allowed to adhere overnight under standard conditions (37 °C, 5% CO_2_). The day after, lentiviruses encoding for the pLSFFV‐Mb‐tdTomato‐IRES‐Blasticidin construct were added in the presence of polybrene (10 µg mL^−1^). After one day of transduction, selection with blasticidin (5 µg mL^−1^) was performed until highly fluorescent cells were FACS sorted.

### Nanoparticles Impact on Immune Cells Viability

Negatively sorted NK and pan T cells (100,000/wells) were treated with increasing concentrations of each NPs type in complete R10 medium. Cells were treated for 48 h under standard conditions (37 °C, 5% CO_2_). Immune cells viability was measured at the endpoint using the CellTiterGlo luminescent cell viability assay (Promega #G7570).

### Immune Cells RNA Extraction and Bulk RNA‐Sequencing—RNA Extraction and Quantification

After 48 h of culture, NK and pan T cells, untreated or treated with NPs at the indicated concentrations, were harvested and washed in ice‐cold PBS. Total RNA was isolated using the RNAeasy kit (Qiagen #74 136) with on‐column DNAse I digestion (Qiagen #79 256) according to manufacturer's instruction. RNA was eluted in 50 µL final volume, and its concentration was assessed with the NanoPhotometer N60 (Implemen). RNA integrity was assessed using a total RNA Pico Kit by Bioanalyzer 2100 Instrument (Agilent Technologies). All samples had RNA integrity numbers above seven.

### Library Preparation and Sequencing

RNA sequencing libraries were generated using the NEBNext Ultra II Directional RNA Library Prep Kit for Illumina, following enrichment of polyadenylated mRNA with the NEBNext Poly(A) mRNA Magnetic Isolation Module (New England Biolabs). Prepared libraries were pooled and sequenced on the Illumina NextSeq 2000 platform using single‐end 100 bp reads, in accordance with the manufacturer's instructions.

### Multidimensional Scaling (MDS) and Differential Expression Analysis

For each sample, quality control was carried out and assessed with the NGS Core Tools FastQC.^[^ ^107^ ^]^ A minimum of 22.4 million sequence reads were aligned to the *Homo sapiens* reference genome hg19 using STAR,^[^ ^108^ ^]^ generating Binary Alignment Map (BAM) files. An abundance matrix was generated based on read counts identified by HTSeq‐count.^[^ ^109^ ^]^ Gene expression counts were normalized with the DESeq2 R package,^[^ ^110^ ^]^ and multidimensional scaling (MDS) was applied to assess relative similarities across the different conditions. The first two principal components were plotted against each other, along with their respective variances explained. Differential expression analysis between the NPs treated conditions and the control was conducted using DESeq2^[^ ^110^ ^]^ package of the Bioconductor framework.^[^ ^111^ ^]^ Volcano plots were constructed based on log_2_ fold change (≥ or ≤ 2) and −log_10_ FDR adjusted *p*‐value (<1.3).

### Erichment Analysis

Gene Ontology (GO) term enrichment analysis was performed using Metascape (http://metascape.org).^[^ ^112^ ^]^ Functional interpretation of gene expression changes was further conducted using Ingenuity Pathway Analysis (IPA, Ingenuity Systems).

### Estimation of Aggregate Expression

Genes related to TNFα signaling via NFκB and IFNγ pathways were curated from the MSigDb Hallmark 2024 database (Table S4, Supporting Information). For each experimental condition, aggregate expression levels of the indicated gene signatures were estimated by first performing a normalization on the cell counts. The normalized counts were then z‐scored by gene (across all the conditions), after which the genes of interest were subsetted and their distribution of z‐scored gene counts visualized as violin plots.

### RT‐qPCR Analysis

Expression of downstreamed regulators was assessed by synthesizing complementary DNA (cDNA) with the High‐Capacity cDNA Reverse Transcription Kit (Applied Biosystems #4 368 814), following the manufacturer's instructions. Quantitative PCR (qPCR) was performed on a QuantStudio 3 using TaqMan assays. Target gene expression levels were normalized to GAPDH, and relative expression changes were calculated using the 2^‐∆∆Ct^ method. TaqMan probes were NFKB1 (Hs00765730_m1), NFKB2 (Hs01028890_g1), RELA (Hs01042014_m1), RELB (Hs00232399_m1), IRF1 (Hs00971965_m1), GAPDH (Hs02786624_g1).

### Whole Proteome Analysis—Protein Extraction and Samples Preparation

After 48 h of culture, NK and pan T cells, untreated or treated with NPs at the indicated concentrations, were harvested and washed in ice‐cold PBS. Immune cells in dry pellets were flash frozen in liquid nitrogen. Cellular pellets were resuspended in 2% SDS, 62.5 mM Tris‐HCl pH = 6.8, and lysed using a water bath sonicator cooled with ice. Protein concentration was estimated using the Biorad DC kit (Hercules). Proteins (2 µg) were prepared using a modified SP3 workflow based on.^[^ ^113^ ^]^ Briefly, proteins were reduced 30 min at 37 °C with dithiothreitol (final conc. 12 mM) and alkylated 30 min, RT, in the dark with iodoacetamide (final conc. 40 mM). SP3 magnetic beads (Sera‐Mag SpeedBeads) were rinsed three times with H_2_O before being added to the sample (ratio 1:10 protein/beads). Acetonitrile (final conc. 50% v/v) was added to precipitate the proteins on the beads, and the samples were incubated for 15 min, RT, with agitation. The beads were washed twice with 200 µL of 80% ethanol and once with 180 µL of acetonitrile before being resuspended in 40 µL of ammonium bicarbonate (100 mM) followed by 5 min sonication in a water bath. Trypsin/Lys‐C was added to achieve a final ratio of 1:10 (enzyme:protein), and the proteins were digested overnight, 37 °C, 600 rpm. Samples were acidified with formic acid to a final conc. of 1% v/v and centrifuged for 10 min at 3500 rpm. The samples were incubated for 10 minutes on the magnetic rack, and the supernatants containing the peptides were transferred to a new plate. Peptide clean‐up was performed on a Bravo AssayMap (Agilent) using 5 µL RP‐C18 cartridges (Agilent) following the manufacturer's instructions.

### nLC‐MS/MS Analysis

After evaporation, peptides were resuspended in H_2_O/ACN/FA (98/2/0.1) and 1/6th of the peptides were injected in randomised order on a nanoAcquity (Waters) – Q‐Exactive HF‐X coupling (Thermo Fisher Scientific). Peptides were separated using a 79 min gradient at a flow rate of 400nL/min. The amount of solvent B (ACN/FA, 99.9/0.1) started at 1%, increased to 8% in 2 min, and then to 35% B in 77 mins. The column was washed by increasing the percentage of B to 90% in 1 min and for 5 min before decreasing to 1% B in 2 min and for 2 min to re‐equilibrate the column. MS analysis was performed using a TOP20 data‐dependent acquisition. The scan range was 375 to 1500m/z with a dynamic exclusion of 40s. For precursor analysis, a resolution of 120000 was used with an AGC target of 3.10^6^ and a maximum injection time of 60 ms. For fragment analysis, the resolution was 15000 with an AGC target of 10^5^, a maximum injection time of 60 ms, and an isolation window of 2m/z.

### Data Treatment and Differential Analysis

Data searches were conducted on a local Mascot server (Matrix Science) against a database comprising all *Homo sapiens* protein entries from the UniProtKB/SwissProt databases, along with common MS contaminants. A tolerance of 5 ppm for precursors and 0.05 Da for fragments was applied. Carbamidomethylation of cysteine residues was defined as a fixed modification, while acetylation of the N‐termini of the proteins and oxidation of methionines were defined as variable modifications. Proline studio^[^ ^114^ ^]^ was used for validation of protein identifications and quantification using a 1% FDR at both the protein and PSM levels. Differential protein expression analysis was performed using Prostar software (v 1.22.6).^[^ ^115^ ^]^ Proteins were retained for analysis only if at least two quantified values were available for one condition. Abundance values were normalized using a quantile centering across the analysis. The imputation of the Partially Observed Value (POV) was realised using Structured Least Square Adaptive (SLSA) imputation, whereas the imputation of the values Missing in an Entire Condition (MEC) was realized using det quantile imputation. Hypothesis testing was conducted using the Limma test, comparing each treatment condition to the control. *P*‐values were adjusted using the Benjamini‐Hochberg procedure to control the false discovery rate (FDR), and results were filtered to maintain an FDR of approximately 1%.

### NK Cells Activation and K562 Killing Assay

For NK cells activation, 40000 K562 target cells were seeded per wells in a U‐bottom 96‐wells plate. Untreated or NPs‐treated NK cells were washed and resuspended at 4.10^6^ cells/mL in complete R10 medium and added at increasing effector‐to‐target ratios (0:1: 0.625:1; 1.25:1; 2.5:1 and 5:1) to K562 cells. NK were co‐incubated with their target cells for 4 h under standard conditions (37 °C, 5% CO_2_). After incubation, the cells were washed in PBS, and non‐viable cells were stained with Fixable Viability Dye‐eFluor450 (eBioscience #65‐0863‐14) for 15 minutes at room temperature in the dark. Aspecific antibodies binding was minimized by treating the cells with TruStain FcX anti‐CD32/CD16 blocking antibody (1:50, BioLegend # 422 301) for 20 min at 4 °C. Then, cells were stained with PE anti‐CD56 (1:100, MiltenyiBiotec #130‐113‐312) and PE‐Vio700 anti‐CD69 (1:50, MiltenyiBiotec #130‐112‐615) and APC anti‐NKG2D (1:50, MiltenyiBiotec #130‐111‐846) for 15 min at 4 °C. Samples were acquired with Attune NxT (Invitrogen) flow cytometer, and data were analyzed using FlowJo v10 Software (ThreeStar). Gating strategy is shown in Figure S6A‐B (Supporting Information).

### NK Cells Conjugates Formation

For NK cells conjugates formation, NK cells were stained with 0.5 µM CellTrace Violet (Invitrogen #C34557) for 15 min under standard conditions (37 °C, 5% CO_2_) in PBS and then washed three times with complete R10 medium. In parallel, K562 target cells were stained with 0.5 µM CellTrace CFSE (Invitrogen #C34554) under the same conditions. Cells were then resuspended at 10^6^ cells/mL in complete R10 medium, mixed at a 1:1 ratio, and pelleted with a short spin before incubation for 20 min under standard conditions (37 °C, 5% CO_2_) to assess conjugate formation rates. Then, unspecific conjugates were removed by pipetting, and cells were fixed with 2% PFA in PBS for 20 min at room temperature. After washing the cells with PBS, conjugate formation was analyzed with Attune NxT (Invitrogen) flow cytometer, and data were analyzed using FlowJo v10 Software (ThreeStar).

### NK Cells Immune Synapse Formation

Conjugates between untreated or NPs‐treated NK cells and K562‐palmitoylated tdTomato target cells at a 2:1 ratio were formed in suspension for 20 min at 37 °C in serum‐free R10 medium. Cells were then gently mixed and transferred to 0.01% poly‐L‐lysine coated 12‐wells ibidi slides (ibidi #81 201). Slides were incubated for 25 min under standard conditions (37 °C, 5% CO_2_) and then fixed with 2% PFA in PBS for 20 min at room temperature. Fixative was removed, and wells were rinsed three times with 150 µl PBS. Cells were permeabilized with 0.1% Triton X‐100 / 2% BSA in 1x PBS for 5 min, and unspecific antibody binding was blocked for 1 h in blocking solution (3% bovine serum albumin‐BSA, 5% FBS, 0.01% Triton X‐100, in PBS). Immunostaining was performed with purified mouse anti‐perforin (10 µg mL^−1^, BioLegend, clone dG9), incubated overnight at 4 °C followed by secondary antibodies (Goat anti‐mouse‐AlexaFluor 647, 1:250, ThermoFisher #A‐21236) and Phalloidin‐iFluor488 (1:1000, Abcam #ab176753) for 1 h at room temperature prior to slide mounting (Fluoromount/DAPI). Background and nonspecific staining controls were used.

Perforin‐stained NK‐K562 conjugates were imaged with a 60X water‐immersion objective on an inverted OIympus Spinning‐disk, and *z*‐series images were acquired with a space of 0.45 µm. Images were processed using ImageJ software (National Institutes of Health). For scoring of cytotoxic granules distance to the immune synapse (based on perforin staining), 20 conjugates between NK cells and K562 target cells were chosen randomly per condition. Distance quantification was performed using the ImageJ macro “Shortest_distance_between_objects” as described in Ref. [116]

### Pan T Cells Activation Analysis

For T cell activation, F‐bottom 96‐wells plate was coated with increasing anti‐CD3 concentrations (ranging from 0.001 to 10 µg mL^−1^, BioLegend, clone OKT3) overnight at 4 °C. Wells were washed once in PBS before adding the cells. Untreated or NPs‐treated pan T cells were washed and resuspended at 10^6^ cells/mL in complete R10 medium, and anti‐CD28 (5 µg mL^−1^, BioLegend, clone 28.2) was added to each condition. 100,000 cells per well were incubated for 24 h under standard conditions (37 °C, 5% CO_2_). After incubation, the cells were washed in PBS, and non‐viable cells were stained with Fixable Viability Dye‐eFluor780 (eBioscience #65‐0865‐14) for 15 min at room temperature in the dark. Aspecific antibody binding was minimized by treating the cells with TruStain FcX anti‐CD32/CD16 blocking antibody (1:50, BioLegend # 422 301) for 20 min at 4 °C. Then, cells were stained with FITC anti‐CD3 (1:100, MiltenyiBiotec #130‐126‐882), PE‐Vio700 anti‐CD69 (1:50, MiltenyiBiotec #130‐112‐615), and Brilliant Violet 421 anti‐OX40 (1:20, BioLegend, clone Ber‐ACT35) for 15 min at 4 °C. Samples were acquired with Attune NxT (Invitrogen) flow cytometer, and data were analyzed using FlowJo v10 Software (ThreeStar).

### Pan T Cells Spreading and F‐Actin Analysis

For T cells spreading, Eight‐wells ibidi slides (ibidi #80 841) were coated with 0.01% poly‐L‐lysine for 45 min at room temperature. After washing in PBS, the coverslips were coated with anti‐CD3 (10 µg mL^−1^, BioLegend, clone OKT3) and rhICAM1‐Fc (2 µg mL^−1^, BioLegend #552 906) overnight at 4 °C. Coverslips were washed once in PBS before adding the cells. Untreated or NPs‐treated pan T cells were washed and resuspended at 10^6^ cells/mL in serum‐free R10 medium for 1 h under standard conditions (37 °C, 5% CO_2_). After 100,000 cells per well were incubated with anti‐CD28 (5 µg mL, BioLegend, clone 28.2) for 25 min under standard conditions (37 °C, 5% CO_2_). Coverslips were then washed once with PBS and fixed with 2% PFA in PBS for 20 min at room temperature. Fixative was removed, and wells were rinsed three times with 150 µl PBS. Cells were permeabilized with 0.1% Triton X‐100 / 2% BSA in 1x PBS for 5 min, and F‐actin was stained by Phalloidin‐iFluor488 (1:1000, Abcam #ab176753) for 1 h at room temperature prior to slide mounting (Fluoromount/DAPI).

Phalloidin‐stained pan T cells were imaged with a 60X water‐immersion objective on an inverted OIympus Spinning‐disk, and *z*‐series images were acquired with a space of 0.45 µm. Images were processed using ImageJ software (National Institutes of Health). To analyze cell spreading on surfaces, only the z‐stack plane corresponding to the contact between T cells and the surface was considered, and a projection of the DAPI stain was used to individualize each cell. Briefly, cell areas were obtained by polygon selection definition and spreading area, as well as F‐actin intensity at the immunological synapse was measured. F‐actin intensity was normalized to the area of each individual T cell.

### Pan T Cells Degranulation Analysis

For T cells degranulation, F‐bottom 96‐wells plate was coated anti‐CD3 (10 µg/mL, BioLegend, clone OKT3) and rhICAM1‐Fc (2 µgmL^−1^, BioLegend #552 906) overnight at 4 °C. Wells were washed once in PBS before adding the cells. Untreated or NPs‐treated pan T cells were washed and resuspended at 10^6^ cells/mL in complete R10 medium, and anti‐CD28 (2 µg/mL, BioLegend, clone 28.2) was added to each condition. 100,000 cells per well were incubated for 1 h under standard conditions (37 °C, 5% CO_2_) in the presence of APC‐conjugated anti‐CD107a (1:50, MiltenyiBiotec #130‐111‐847). After 1 h, protein transport inhibitor cocktail (1:500, eBioscience) was added in each well and cells were incubated for an additional 4 h under standard conditions (37 °C, 5% CO_2_). After incubation, the cells were washed in PBS, and non‐viable cells were stained with Fixable Viability Dye‐eFluor780 (eBioscience #65‐0865‐14) for 15 min at room temperature in the dark. Aspecific antibody binding was minimized by treating the cells with TruStain FcX anti‐CD32/CD16 blocking antibody (1:50, BioLegend # 422 301) for 20 min at 4 °C. Then, cells were stained with VioBright B515 anti‐CD3 (1:50, MiltenyiBiotec #130‐126‐882), Pacific Blue anti‐CD4 (1:100, BioLegend, clone RPA‐T4) and PE anti‐CD8 (1:100, BioLegend, clone RPA‐T8) for 15 min at 4 °C. Samples were acquired with Attune NxT (Invitrogen) flow cytometer and data were analyzed using FlowJo v10 Software (ThreeStar).

### Cytokine Measurement

Supernatants from 96‐well plates were aliquoted after 4 h of NK‐K562 cells co‐incubated or after 24 h of pan T cells activation and stored at −20 °C. Cytokines were quantified by custom LEGENDplex (5‐plex, BioLegend # 740 510) with the Attune NxT Flow Cytometer. Cytokine assays were analyzed using the LEGENDplex software Qognit (https://legendplex.qognit.com).

### Statistics

Statistical analyses were conducted using GraphPad Prism 9.5. Gaussian distribution of the data was assessed with the Shapiro‐Wilk test. Depending on the number of groups compared, either Student's t‐test or One‐way ANOVA, followed by the Benjamini‐Hochberg original false discovery rate (FDR) correction, was applied. The significance threshold α was set at 0.05, and significance levels are indicated as *p < 0.05; **p < 0.01, ***p < 0.001, ****p < 0.0001. All the data were presented as median ± standard deviation.

### Data Availability

Raw RNA‐seq data had been deposited in the EMBL‐EBI ArrayExpress archive (accession number E‐MTAB‐14550). Complete proteomics dataset had been deposited to the ProteomeXchange Consortium via the PRIDE partner repository^[^ ^117^ ^]^ (accession number PXD056695). All the other data are available within the article and its Supplementary Information. Raw data are accessible through reasonable requests to the corresponding authors.

## Conflict of Interest

A.D., O.T., and F.L. are shareholders of NH Theraguix who is translating to the clinic Gd‐NPs. The other authors declare that they have no conflict of interest.

## Author Contributions

The conceptualization of the study was carried out by V.M., O.L., J.G.G., and A.D. Methodology was developed by V.M. and O.L., while the investigation was conducted by V.M., M.C.D., J.B., A.L., A.P., and C.M. Resources were provided by M.B., S.G., L.J.C., F.L., and O.T. Formal analysis was performed by V.M., O.L., A.D., M.C.D., L.B., S.H., A.P., T.S., A.M., M.R., C.C., and R.C. The original draft of the manuscript was written by V.M., O.L., J.G.G., and A.D., with review and editing contributions from V.M., O.L., C.C., R.C., L.J.C., F.L., O.T., J.G.G., and A.D. Supervision was provided by C.C., R.C., J.G.G., and A.D., while funding acquisition was handled by J.G.G. and A.D.

## Supporting information



Supporting Information

## Data Availability

The data that support the findings of this study are available from the corresponding author upon reasonable request.
