# Supplementary material for: Nanomaterials Trigger Functional Anti‐Tumoral Responses in Primary Human Immune Cells
Source: Adv Sci (Weinh). 2025 Jul 12;12(39):e05729. doi: 10.1002/advs.202505729 (PMC12533298; doi:10.1002/advs.202505729)
Supplement: Supplementary file 1 — Supporting Information [file ADVS-12-e05729-s001.docx]

**Nanomaterials trigger functional anti-tumoral responses in primary human immune cells**

Vincent Mittelheisser^1-6^, Olivier Lefebvre^1-5^, Mainak Banerjee^5-7^, Shayamita Ghosh^5-7^, Amandine Dupas^1-5^, Marie-Charlotte Diringer^5-7^, Juliette Blumberger^5-7^, Louis Bochler^1-5^, Sébastien Harlepp^5-7^, Annabel Larnicol^1-5^, Angélique Pichot^2-4,8^, Tristan Stemmelen^2-4,8^, Anne Molitor^2-4,9^, Chloé Moritz^9^, Christine Carapito^9^, Raphaël Carapito^2-4,8,10^, Loïc J. Charbonnière^11^, François Lux^12,13^, Olivier Tillement^12^, Jacky G. Goetz^1-5,*^ and Alexandre Detappe^5-7,11,*^

^1^Tumour Biomechanics lab, Strasbourg, France.

^2^INSERM UMR_S1109, Strasbourg, France.

^3^Université de Strasbourg, Strasbourg, France.

^4^Fédération de Médecine Translationnelle de Strasbourg (FMTS), Strasbourg, France.
^5^Équipe Labellisée Ligue Contre le Cancer.

^6^Nanotranslational lab, Institut de cancérologie Strasbourg Europe.

^7^Strasbourg Drug Discovery and Development Institute (IMS), Strasbourg, France.

^8^Plateforme GENOMAX, Institut thématique interdisciplinaire (ITI) de Médecine de Précision de Strasbourg Transplantex NG, Faculté de Médecine.

^9^Laboratoire de Spectrométrie de Masse BioOrganique (LSMBO), IPHC UMR 7178, CNRS, Université de Strasbourg, Infrastructure nationale ProFI FR 2048, Strasbourg, France.

^10^Service d'Immunologie Biologique, Plateau Technique de Biologie, Pôle de Biologie, Nouvel Hôpital Civil, Hôpitaux Universitaires de Strasbourg, 1 Place de l'Hôpital, 67091, Strasbourg, France.

^11^Institut Pluridisciplinaire Hubert Curien CNRS UMR 7178, Strasbourg, France.

^12^Institut Lumière Matière, CNRS UMR 5306, Université Claude Bernard – Lyon 1, Villeurbanne, France.

^13^Institut Universitaire de France (IUF), Paris, France.

***Correspondence and co-last authors**

Jacky G. Goetz, [jacky.goetz@inserm.fr](mailto:jacky.goetz@inserm.fr)

Tumour Biomechanics lab, INSERM UMR_S1109, Centre de Recherche en Biomédecine de Strasbourg

1 rue Eugène Boeckel, 67000 Strasbourg.

Web: [www.goetzlab.fr](http://www.goetzlab.com), @GoetzJacky

Alexandre Detappe, a.detappe@icans.eu

Institut de Cancérologie Strasbourg

3, rue de la porte de l’Hôpital, 67000 Strasbourg France

Web: www.detappelab.com, @AlxDetappe


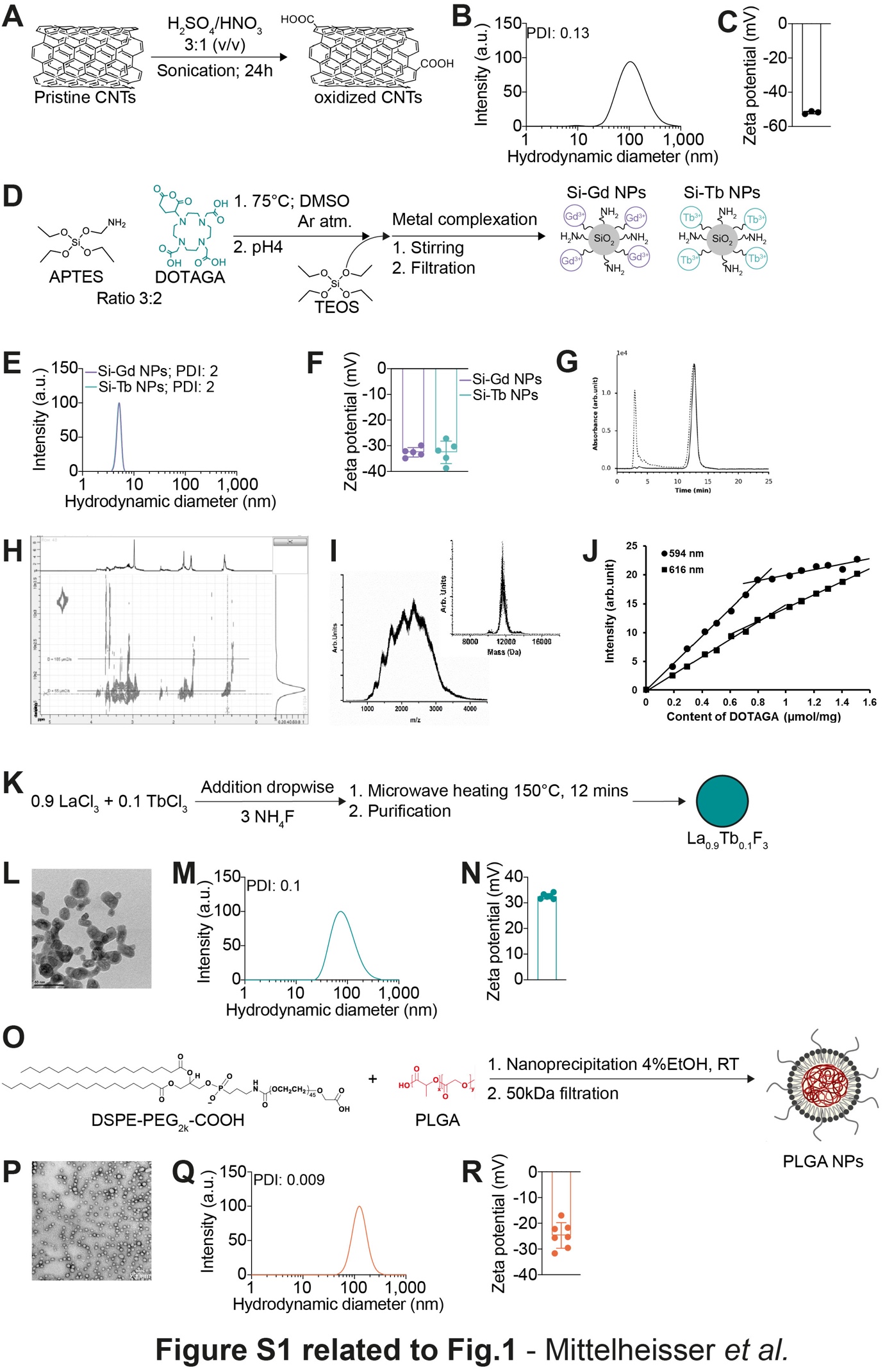


**Supplementary Figure S1. Nanomaterials synthesis and characterization. A.** Prisitin CNTs were shortened under strong acid conditions (H_2_SO_4_/HNO_3_ 3:1) and sonication for 24h to generate a high amount of carboxylic groups. **B-C.** oxidated CNTs size and charge were measured by DLS. **D.** Ultrasmall polysiloxane-based gadolinium (Si-Gd) NPs were synthesized by a top-down method from core (gadolinium oxide) shell (polysiloxane) NPs whereas ultrasmall polysiloxane-based terbium (Si-Tb) NPs were synthesized with a bottom-up one pot synthesis. **E-F.** Si-Gd NPs and Si-Tb NPs size and charge were measured by DLS. **G**. Chromatograms of polysiloxane-based NPs before (dashed line) and after (solid line) tangential filtration. Absorbance was measured at 295 nm. **H**. NMR DOSY spectrum of polysiloxane nanoparticles. **I**. ESI-MS spectrum of polysiloxane nanoparticles. **J**. Europium titration of DOTAGA in polysiloxane nanoparticles. **K.** Terbium fluoride (Tb) NPs were synthetized by dropwise addition of 0.9 LaCl_3_ and 0.1 TbCl_3_ to 3 NH_4_F followed by heating at 150°C for 12 mins and purification. **L.** Transmission electron microscopy of Tb NPs. **M-N.** Tb NPs size and charge were measured by DLS. **O.** PLGA NPs were synthetized by self-assembly of DSPE-PEG_2k_-COOH and PLGA through a one-step nanoprecipitation. **P.** Transmission electron microscopy of PLGA NPs. **Q-R.** PLGA NPs size and charge were measured by DLS.


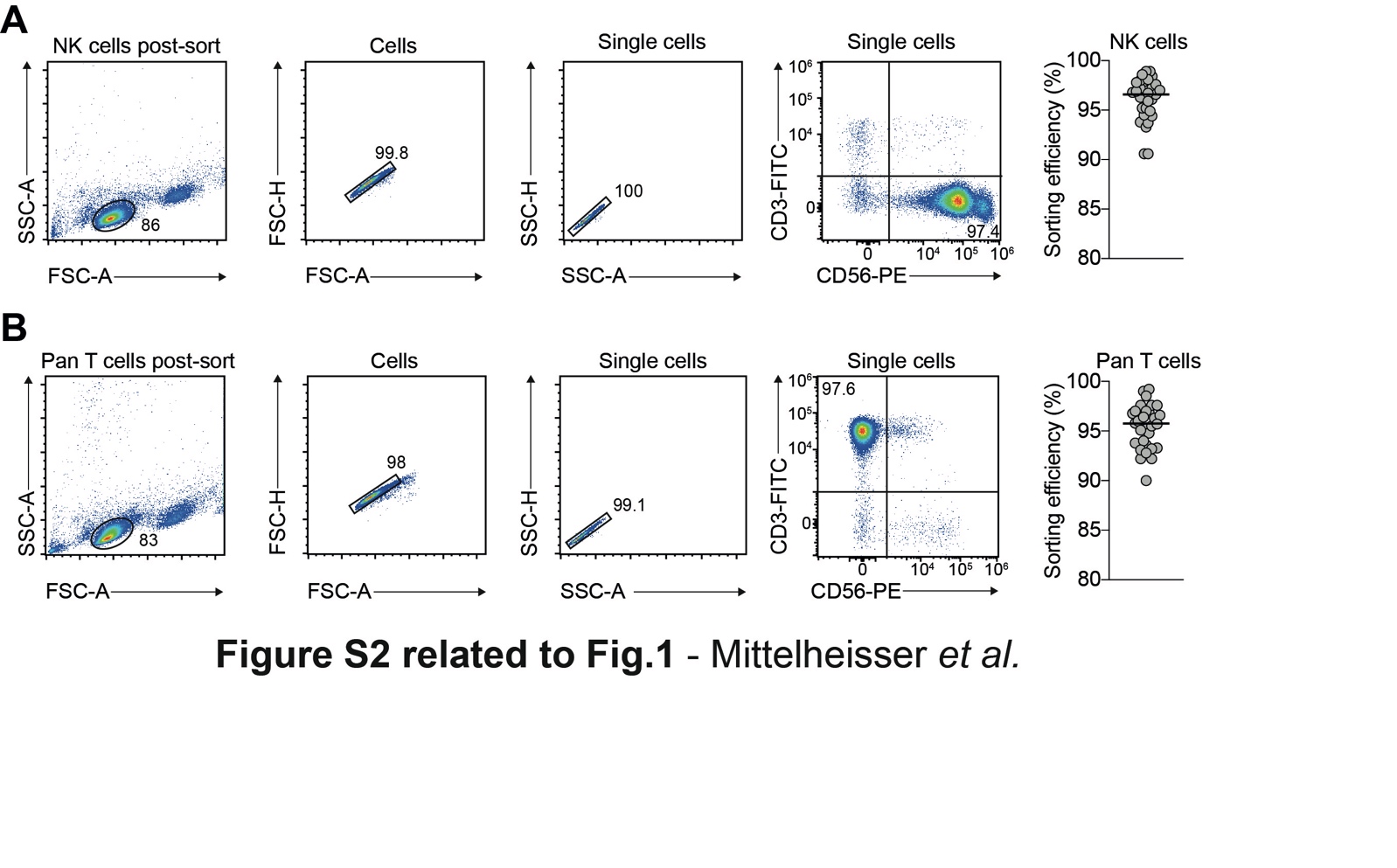


**Supplementary Figure S2. NK cells and pan T cells isolation.** NK cells and pan T cells immunophenotyping. **A.** Left: Gating strategy for assessment of NK cells sorting efficiency. Right: Percentage of pure CD3^-^ CD56^+^ NK cells after sorting. Data are representative of 31 independent donors. Median of the data is presented. **B.** Left: Gating strategy for assessment of pan T cells sorting efficiency. Right: Percentage of pure CD3^+^ pan T cells after sorting. Data are representative of 30 independent donors. Median of the data is presented.


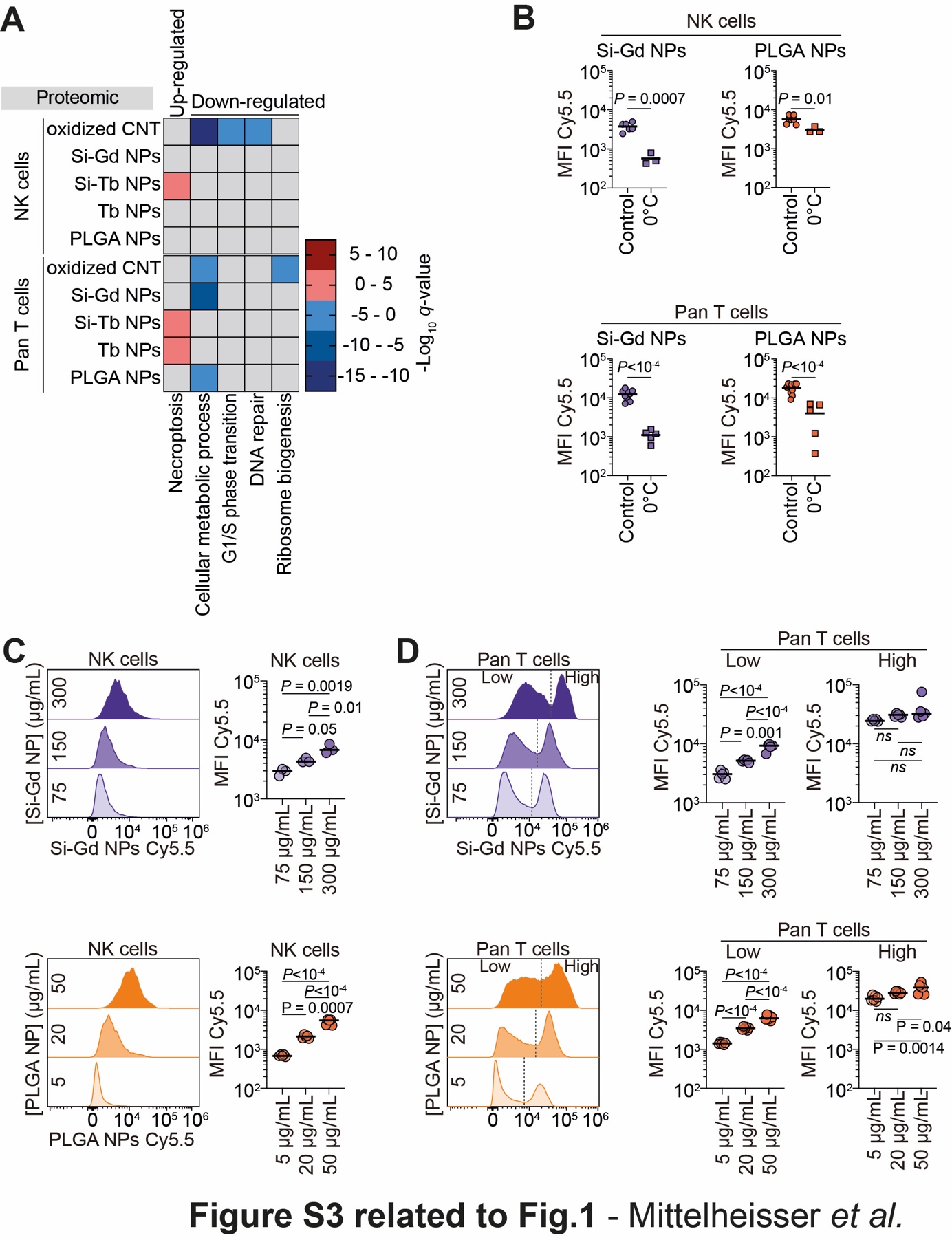


**Supplementary Figure S3. Internalization and biological impact of nanomaterials on NK and pan T-cells A.** Heat-map showing -Log_10_(*q*-value) for up-regulated and down-regulated gene ontology terms in NK cells and pan T cells upon 48h treatment with different nanomaterials. **B**. Quantification of the mean fluorescent intensity signal of the Cyanine5.5-labelled nanomaterials to assess their internalization metabolism dependency in NK cells (upper panel) and pan T cells (lower panel) after 8h co-incubation at 37°C (Control) or on ice (0°C). Data are representative of 3 to 11 independent donors and analyzed by a Student’s t-test or a Mann-Whitney test (PLGA NPs in NK cells) after assessment of their gaussian distribution by Shapiro-Wilk test. **C.** Flow-cytometry assessment of nanomaterials internalization dose-dependency in NK cells after 48h co-incubation at 37°C. Left: Representative histograms of the relative internalization of Si-Gd NPs (upper panel) and PLGA NPs (lower panel). Right: Quantification of the mean fluorescent intensity signal of the Cyanine5.5-labelled nanomaterials. Data are representative of 3 to 6 independent donors and analyzed by a One-way ANOVA test with original FDR method of Benjamini-Hochberg after assessment of their gaussian distribution by Shapiro-Wilk test. **D.** Flow-cytometry assessment of nanomaterials internalization dose-dependency in pan T cells after 48h co-incubation at 37°C. Left: Representative histograms of the relative internalization of Si-Gd NPs (upper panel) and PLGA NPs (lower panel). Right: Quantification of the mean fluorescent intensity signal of the Cyanine5.5-labelled nanomaterials in low and high internalizing population as gated on the histograms. Data are representative of 5 to 6 independent donors and analyzed by a One-way ANOVA test with original FDR method of Benjamini-Hochberg after assessment of their gaussian distribution by Shapiro-Wilk test.


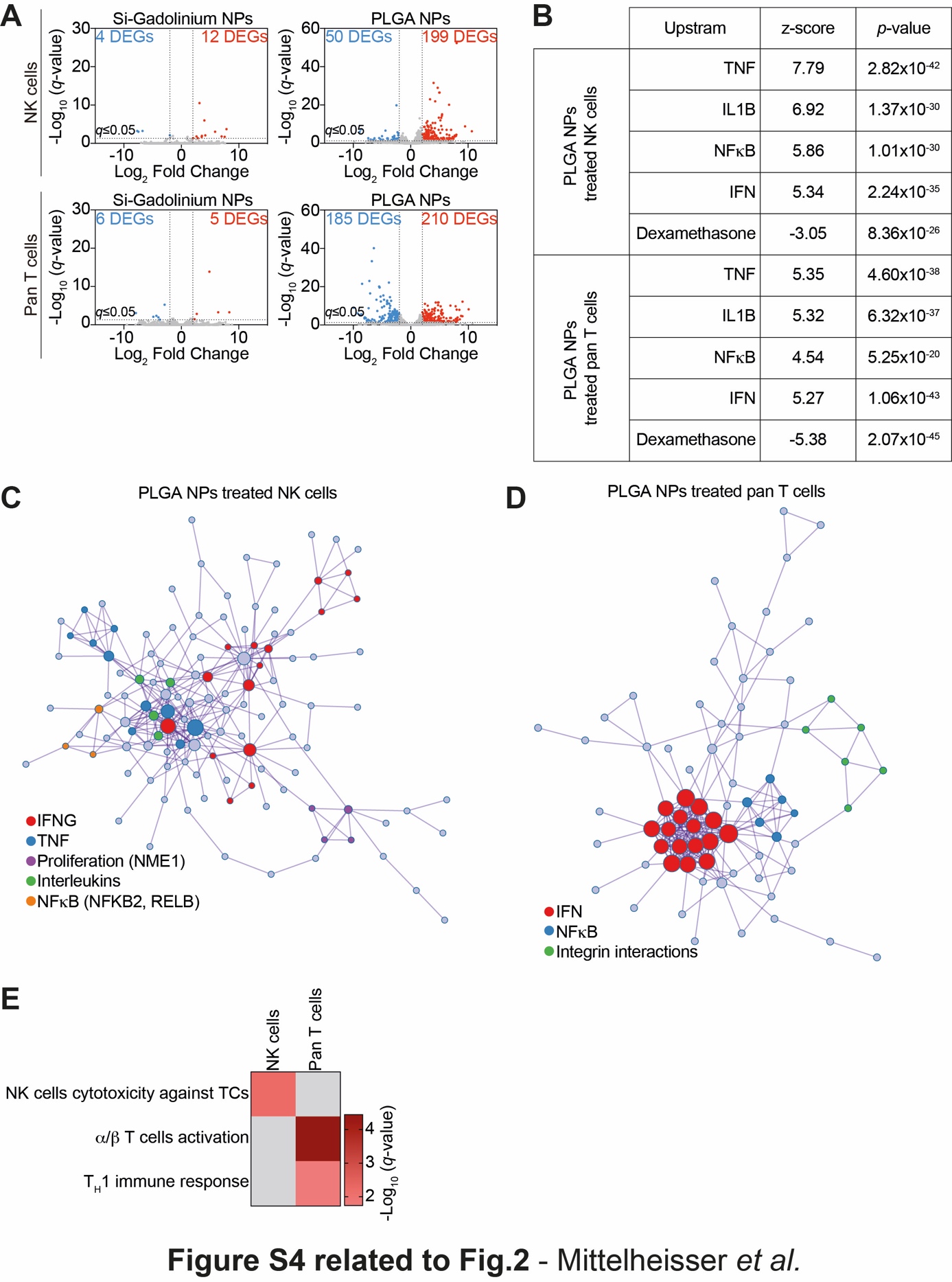


**Supplementary Figure S4.** **Transcriptomic profiling and network analysis of NK and pan T-cells treated with Si-Gd and PLGA nanoparticles.** Volcano plot display differentially expressed genes between Si-Gd NPs- or PLGA NPs-treated NK cells (upper panel) or pan T cells (lower panel). Threshold -Log10 (*q*-value) ≤ 1.3 and fold-change > 2. *q*-value = adjusted *p-*value. **B.** Upstream analysis showing the top upstream molecules predicted to cause the observed gene expression changes. **C-D.** STRING predicted protein-protein interactions based on the upregulated genes in PLGA NPs-treated NK cells (**B**) and pan T cells (**C**) and their functional annotations. **E.** Heat-map showing -Log10 (*q*-value) of immune cells specific gene ontology terms assignment from upregulated genes by PLGA NPs treatment of NK cells and pan T cells.


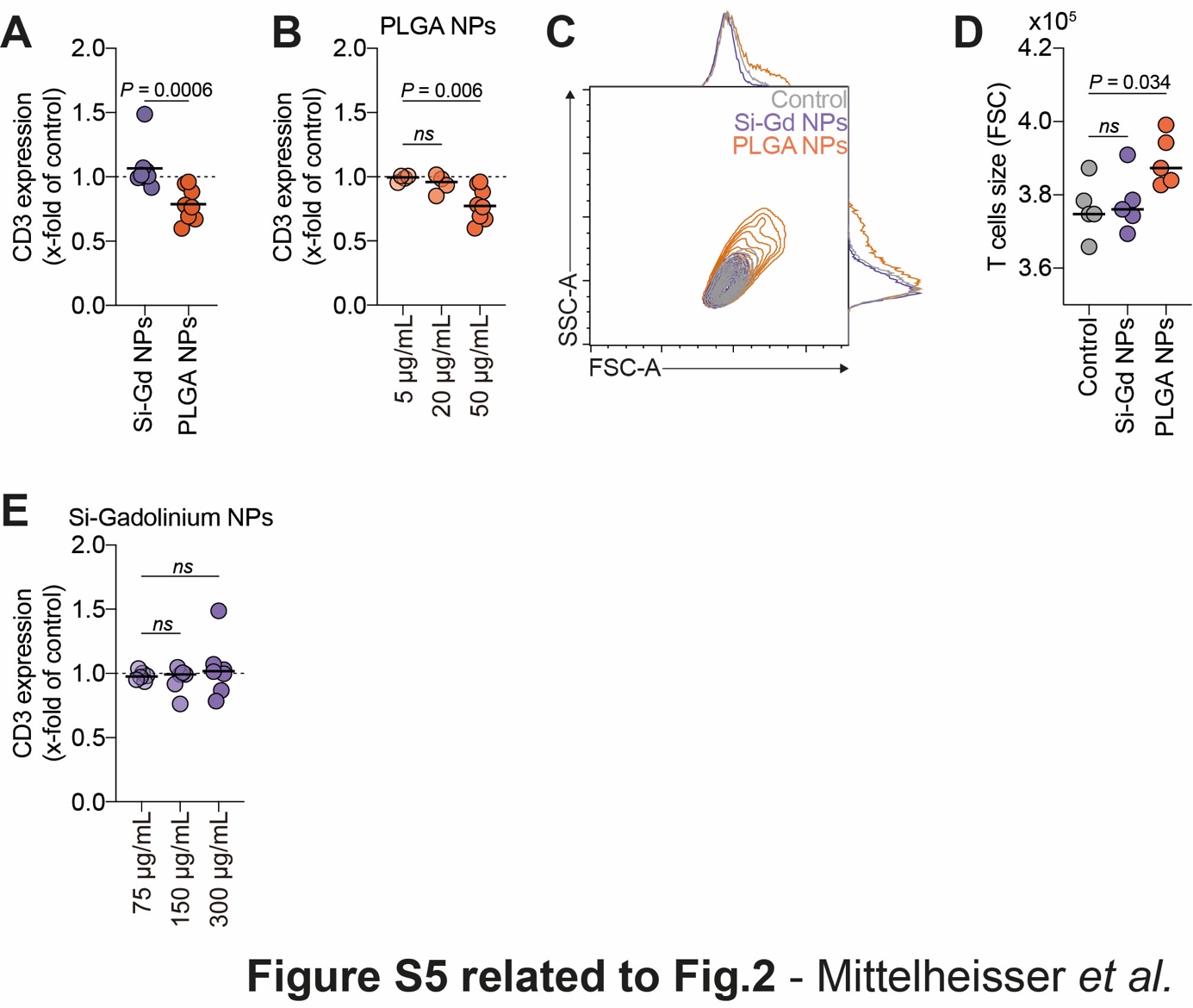


**Supplementary Figure S5.** **Dose-dependent modulation of CD3 expression and morphological changes in pan T-cells upon treatment with Si-Gd and PLGA nanoparticles.** Quantification of CD3 expression at the surface of pan T cells after 48h of treatment with Si-Gd NPs or PLGA NPs. Data are represented as fold change relative to untreated control. Data are representative of 8 independent donors and analyzed by a Mann-Whitney test after assessment of their gaussian distribution by Shapiro-Wilk test. **B.** Quantification of CD3 expression at the surface of pan T cells after 48h of treatment with increasing concentrations of PLGA NPs. Data are represented as fold change relative to untreated control. Data are representative of 4 to 8 independent donors and analyzed by a Brown-Forsythe and Welch's ANOVA test with original FDR method of Benjamini-Hochberg after assessment of their gaussian distribution by Shapiro-Wilk test. **C.** Representative flow cytometry contour plots of the pan T cells size (FSC) and structure (SSC) obtained by flow cytometry after Si-Gd NPs and PLGA NPs treatment of 48h. **D.** Quantification of pan T cells size based on (**C**) flow cytometry experiments after 48h of treatment with increasing concentrations of PLGA NPs. Data are representative of 5 independent donors and analyzed by a One-way ANOVA test with original FDR method of Benjamini-Hochberg after assessment of their gaussian distribution by Shapiro-Wilk test. **D.** Quantification of CD3 expression at the surface of pan T cells after 48h of treatment with increasing concentrations of Si-Gd NPs. Data are represented as fold change relative to untreated control. Data are representative of 6 to 8 independent donors and analyzed by a Kruskal-Wallis test after assessment of their gaussian distribution by Shapiro-Wilk test.


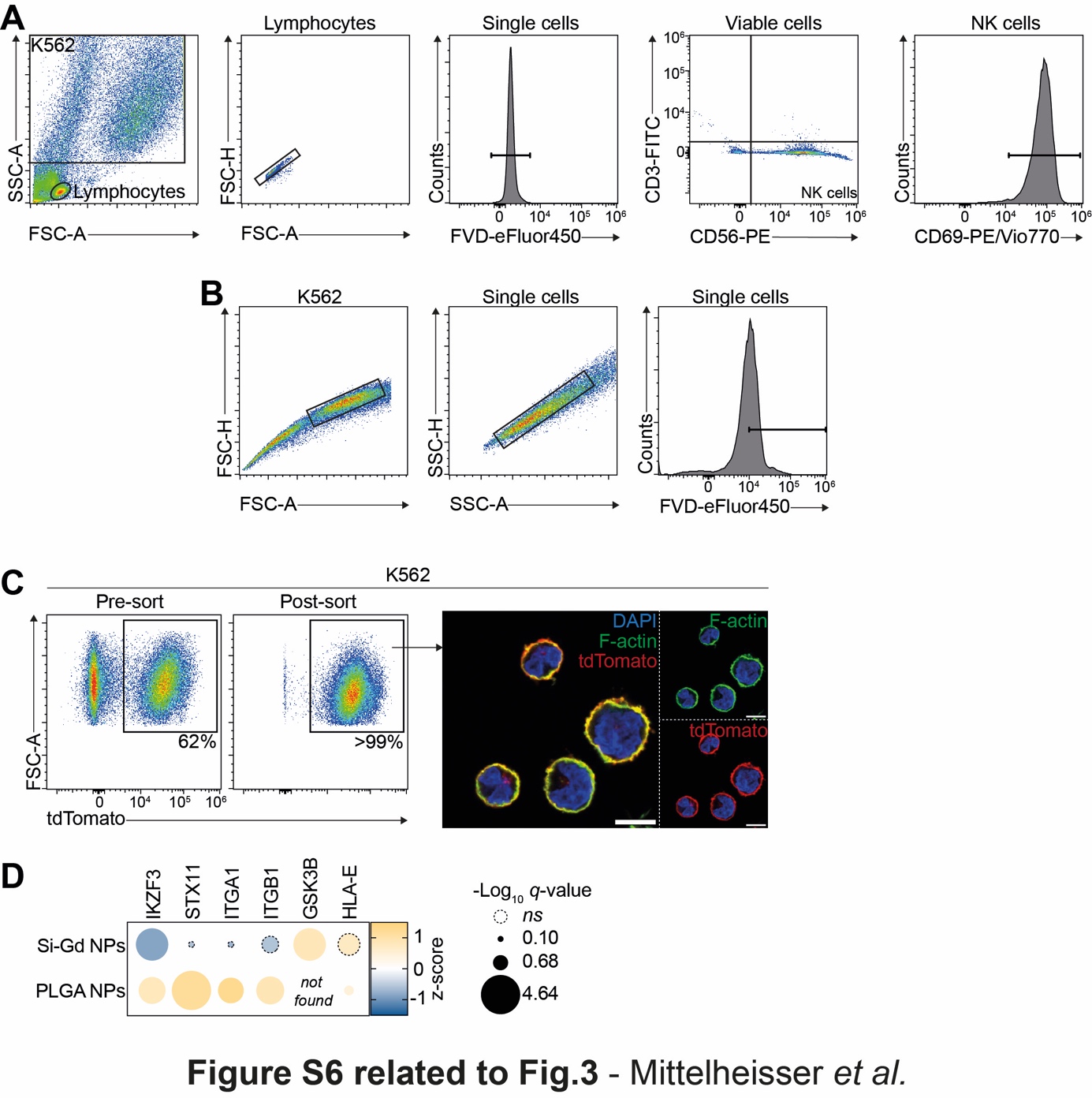


**Supplementary Figure S6. Functional assessment and transcriptomic profiling of NK cell activation and cytotoxicity against K562 cells. A.** Gating strategy for assessment of NK cells activation levels. **B.** Gating strategy for assessment of NK cells-induced K562 lysis. **C.** Generation of a K562 transgenic cells expressing palmitoylated-tdTomato at their surface. Right: Representative pseudo-color flow cytometry plot before and after fluorescence-activated cells sorting for high tdTomato fluorescence. Left: Representative confocal micrographs of K562-tdTomato cells. In green Phalloidin-iFluor488, in red palmitoylated-tdTomato, in blue nuclei (DAPI). Scale bar = 10µm. **D.** Bubble plot showing Z-score values for genes/proteins expression associated with NK cells functions. Bubble size represents -Log_10_(*q*-value). Dashed lines bubbles correspond to non-significantly deregulated genes/proteins.
